# Innovative Use of an Injectable, Self-Healing Drug-Loaded Pectin-Based Hydrogel for Micro- and Supermicro-Vascular Anastomoses

**DOI:** 10.1021/acs.biomac.4c00102

**Published:** 2024-06-27

**Authors:** Banu Kocaaga, Tugce Inan, Nesrin İsil Yasar, Can Ege Yalcin, Fethiye Aylin Sungur, Ozge Kurkcuoglu, Anil Demiroz, Hasan Komurcu, Osman Kizilkilic, Servet Yekta Aydin, Ovgu Aydin Ulgen, Fatma Seniha Güner, Hakan Arslan

**Affiliations:** 1 Department of Chemical Engineering, Istanbul Technical University, Maslak, 34469 Istanbul, Turkey; 2 Informatics Institute, Computational Science and Engineering Division, Istanbul Technical University, Maslak, 34469 Istanbul, Turkey; 3 Cerrahpasa Medical Faculty, Department of Plastic, Reconstructive and Aesthetic Surgery, Istanbul University-Cerrahpasa, Istanbul 34089, Turkey; 4 Department of Plastic, Reconstructive and Aesthetic Surgery, Balat Or-Ahayim Hastanesi, Istanbul 34087, Turkey; 5 Cerrahpasa Medical Faculty, Department of Interventional Radiology, Istanbul University-Cerrahpasa, Istanbul 34098, Turkey; 6 Cerrahpasa Medical Faculty, Department of Pathology, Istanbul University-Cerrahpasa, Istanbul 34098, Turkey; 7 Sabancı University Nanotechnology Research and Application Center, Istanbul 34956, Turkey

## Abstract

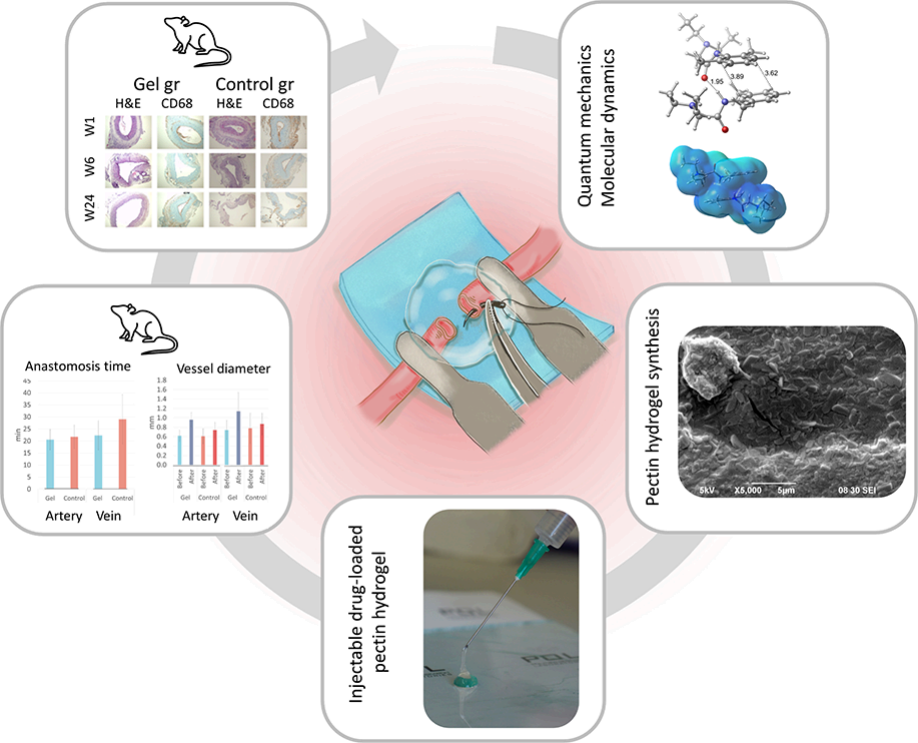

Microvascular surgery plays a crucial role in reconnecting micrometer-scale vessel ends. Suturing remains the gold standard technique for small vessels; however, suturing the collapsed lumen of microvessels is challenging and time-consuming, with the risk of misplaced sutures leading to failure. Although multiple solutions have been reported, the emphasis has predominantly been on resolving challenges related to arteries rather than veins, and none has proven superior. In this study, we introduce an innovative solution to address these challenges through the development of an injectable lidocaine-loaded pectin hydrogel by using computational and experimental methods. To understand the extent of interactions between the drug and the pectin chain, molecular dynamics (MD) simulations and quantum mechanics (QM) calculations were conducted in the first step of the research. Then, a series of experimental studies were designed to prepare lidocaine-loaded injectable pectin-based hydrogels, and their characterization was performed by using Fourier transform infrared spectroscopy (FT-IR), scanning electron microscopy (SEM), and rheological analysis. After all the results were evaluated, the drug-loaded pectin-based hydrogel exhibiting self-healing properties was selected as a potential candidate for *in vivo* studies to determine its performance during operation. In this context, the hydrogel was injected into the divided vessel ends and perivascular area, allowing for direct suturing through the gel matrix. While our hydrogel effectively prevented vasospasm and facilitated micro- and supermicro-vascular anastomoses, it was noted that it did not cause significant changes in late-stage imaging and histopathological analysis up to 6 months. We strongly believe that pectin-based hydrogel potentially enhanced microlevel arterial, lymphatic, and particularly venous anastomoses.

## 1 Introduction

Microvascular tissue transfers are widely practiced in the field of reconstructive surgery, where surgical expertise and skill are required for this technically challenging and time-consuming field.^1^ The most severe complication is flap failure, which mostly arises from the development of thrombi in the vicinity of the anastomosis site, especially around the veins, resulting in disrupted blood flow to the recipient site^2^ and leading to the loss of tissue.^3^ Despite all technical disadvantages, classical micro suturing techniques remain the gold standard in microsurgery, not only because of their availability and low cost but also because of their effectiveness in overcoming vessel diameter mismatches.^4^ In fact, microsurgical aids that can help overcome the difficulties associated with ultrasmall vessel anastomosis are of the utmost importance to further increase the success of these complex procedures.^5,6^ For this purpose, materials such as hydrogels, metal stents, or tubes for stent placement inside the vessel walls have been developed for microvascular anastomosis with the help of tissue adhesives.^7−9^

Hydrogels mimic the wet environment of natural cells due to their three-dimensional cross-linked structure, insolubility in biological fluids, and high fluid retention capacity, and are therefore widely used as biomaterials. Injectable hydrogels based on synthetic and natural polymers^10−14^ form a highly promising category of gels with tunable structures and stimulus-sensitive biodegradation properties. They find extensive use in various biomedical applications such as tissue engineering, wound healing, implants, controlled delivery of drugs, genes, and proteins, cancer treatment, sensor technology^15^ as well as microvascular anastomosis.^16−20^ So far, only two types of hydrogels have been used to facilitate microvascular anastomosis.^7,16,21^ These are either poloxamer or peptide-based in nature. Poloxamer 407 in gel form together with cyanoacrylate tissue adhesive was employed in a sutureless and atraumatic vascular anastomosis technique to conduct microvascular anastomosis.^7^ Although using readily available materials for gel formation, this innovative approach held great promise for microvascular surgery and has led to a series of publications on Poloxamer 407 and cyanoacrylate^22,23^ and peptide-based^16^ hydrogels. Positive outcomes were achieved for these hydrogels; however, several material and technical challenges were limiting their clinical usage. For instance, an external heat source was required to raise the temperature by approximately 40 °C^7^ during the surgical procedure to create a gel media for thermoreversible Poloxomer 407, which is not practical in clinical application. In addition, in the context of microvascular surgery, the novel approach was exclusively employed in arteries but not in veins.^7,16^ Most importantly, the diameter mismatch remained unresolved, rendering it unsuitable for clinical use.^7^ For the peptide-based gel, no additional external stimuli were necessary for gelation; the liquid within the syringe gelled as it exited the tip under the injection’s pressure.^16^ After anastomosis, it passed from the environment to liquid form with the help of UV light for 2 min resuming the blood flow. However, the application was successful only for arteries below 1 mm and did not work in veins. The study also lacked data showing detailed anastomotic success, patency, and duration, important for long-term results.

Technically, vein anastomosis is more difficult than artery anastomosis in microsurgery operations and the risk of thrombus is higher.^24^ The limited availability of microsurgical materials that can aid in venous anastomosis to the same degree as arterial anastomosis in the literature has motivated us to find a unified solution to the anastomosis problems that arise in both arteries and veins. Here, we report the development and *in vivo* application of injectable pectin-based hydrogels for micro- and supermicro-vascular anastomosis, offering a better alternative over poloxamers and peptide-based hydrogels readily coming with limitations. The pectin-based matrix hydrogel was developed to be applied in arteries and especially in veins, which pose the most difficulty in supermicro-vascular anastomosis due to their thinner and flexible nature as opposed to arteries.

Pectin is a natural nontoxic water-soluble polysaccharide and consists mainly of α-D-galacturonic acid residues as well as methoxy and ester groups in different amounts depending on its source.^25^ Low-methoxy pectin (degree of esterification <50%) can form a three-dimensional network gel with a divalent cation such as Ca^2+^ called an “egg-box structure”. This unique structure especially plays a crucial role in controlled drug delivery.^26−31^ The physical properties of pectin hydrogel can be easily altered for specific applications such as making it more flexible while improving structural integrity with the addition of 2-thiobaributric acid,^31^ or improving its oxygen permeability with the addition of zeolite.^32^ It is also possible to chemically modify pectin with aldehyde and to synthesize an injectable, biodegradable, and self-healing pectin-based hydrogel using poly(*N*-isopropylacrylamide) bearing acylhydrazide groups.^33^ Modifying the biomaterials for specific mechanical properties or drug-release behaviors is a great challenge consuming time and money during optimization. At this point, computational techniques such as Molecular Dynamics simulations can reveal the extent of interactions between the molecular entities forming the whole system at the atomistic scale, and shed light on its macroscale behavior, such as predicting the ability of nanosystems for controlled drug delivery.^28,34,35^ In addition, the Density Functional Theory computations for polymer-based drug delivery systems can explain the atom–atom interactions more precisely, which in turn tremendously help the design and the characterization of the drug delivery systems.^36^

To enhance the efficacy of the micro- and supermicro-vascular anastomosis, we aimed for a high light permeability of the pectin hydrogels to ensure unobstructed vision during suturing. Moreover, the hydrogels had to be designed for an effective release of lidocaine to prevent vasospasm during the operation. Furthermore, to maintain high transparency and thus prevent light refraction caused by deformations of a needle during operation, the pectin matrix had to have a self-healing property. It would be an advantage to be able to incorporate these properties into the hydrogel with a simple procedure to ensure its practical usage in clinical application. With this motivation, our research was structured into three distinct phases; (i) computational studies to understand the extent of interactions between drug and pectin chain to achieve an effective drug release, (ii) experimental studies to prepare and characterize the injectable pectin-based hydrogel, and (iii) *in vivo* studies to determine the performance of the hydrogel during operation. Overall, this article presents the preparation conditions and characterization outcomes of self-healing injectable pectin hydrogels loaded with lidocaine, highlighting their potential for researchers in the microsurgical field. To the best of our knowledge, this is the first study to formulate a versatile pectin-based hydrogel for microvascular anastomoses while overcoming the challenges with peptide and poloxamer-based approaches.

## 2 Materials and Methods

### 2.1 Computational Methods

The pectin chain was modeled as a 22-unit long poly galacturonic acid (PGAL) oligomer using Glycan Reader^37^ with the CHARMM36 force field.^38^ This length was found to be sufficient to provide different orientations of lidocaine molecules establishing nonbonded interactions with the chain, similar to our previous study.^28^ The three-dimensional structure of lidocaine was downloaded from PubChem databank^39^ and then parametrized in the CHARMM-GUI Ligand Reader server^40^ using the CHARMM General Force Field.^38^ The carboxyl groups of PGAL were deprotonated (p*K*_a_ of 3.7^41^), and the lidocaine was protonated (p*K*_a_ of 7.7^42^) to mimic the experimental conditions with the model structures.

Molecular dynamics (MD) simulations were conducted to generate conformers of lidocaine-lidocaine and lidocaine-PGAL dimers for the quantum mechanics (QM) calculations to determine their interaction energies. With this purpose, three systems were prepared for the MD simulations; (i) neutral lidocaine in CaCl_2_ solution, (ii) protonated lidocaine in CaCl_2_ solution, and (iii) protonated lidocaine and PGAL in water to mimic different pH values in experimental studies. Simulation systems were prepared using the user interface of Visual Molecular Dynamics (VMD).^43^ The software used for the MD simulations was Nanoscale Molecular Dynamics (NAMD) version 2.13.^44^ In the systems, ten or 12 copies of lidocaine molecules were employed along with TIP3P water molecules, and the net charge of the simulation box was neutralized with the addition of Na^+^ and Cl^–^ ions. These systems represent a dilute concentration of lidocaine solutions that was found to be appropriate for monitoring lidocaine-lidocaine and lidocaine-PGAL chain interactions. The details of the simulated systems are given in Supplementary Table S1. A minimization of 10,000 steps of conjugate gradient was conducted to remove the steric clashes. An NVT simulation of 250 ps long was then performed to equilibrate the system at 298 K. The production simulation was carried out for 50 ns with NPT ensemble at 1 atm and 298 K. The time step was set to 2 fs using the SHAKE algorithm^45^ 1,4 α–α glycosidic bond of PGAL oligomer was constrained during the simulations to represent a segment from the pectin hydrogel, which is expected to provide different interaction sites for lidocaine molecules. CHARMM36 force field was employed to simulate the systems.^38^ The system’s temperature and pressure were controlled with Langevin thermostat and modified Nose-Hoover barostat. Particle Mesh Ewald (PME)^46^ was applied with a cutoff of 12 Å to calculate long-range interactions. The simulations were repeated twice with different initial velocities. After the energy and temperature profiles of the systems were controlled to reach equilibrium values, various conformers of lidocaine-lidocaine and lidocaine-PGAL describing their interactions were selected.

The types of nonbonded interactions between molecule pairs were elucidated with the radial distribution function g(r) using the radial distribution function plugin of VMD software^47^ as,
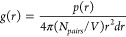
1
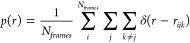
2

Here, r is the distance between the particle pair, *V* is the total volume of the system, and *N*_*pairs*_ is the number of unique pairs of atoms from two sets of selection. *N*_*frames*_ is the number of frames, *r*_*ijk*_ is the distance between atom *j* and atom *k* for the *i*^*th*^ frame, and δ is the Dirac delta function. The calculations were done over 500 frames for each run, i.e. for every 100 ps of 50 ns long simulations.

QM calculations were performed for the geometry optimization and determination of the interaction energies for the selected complexes in implicit water. The initial structures were selected from the snapshots of the MD simulations for QM calculations. Here, at least ten random snapshots were taken from the production runs of each system; the snapshots were carefully inspected and coordinates of at most five different interacting pairs, i.e. with different orientations and distances were saved.

QM calculations were carried out at the ωB97xD theory employing 6-311++G(d,p) basis set at the temperature of 298.15 K.^48^ The interaction energies can be calculated by the supramolecular approach, where the interaction energy of the complex is obtained by subtraction of the energies of the monomers from the total energy of the complex. This resulted in an artificial stabilization of the molecular complex due to the overlapping of basis functions and this is known as the basis set superposition error (BSSE). The BSSE was calculated successfully for the gas-phase systems using the counterpoise correction (CP) method with the equation
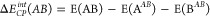
3proposed by Boys and Bernardi.^49^ The superscript *AB* on monomers indicates the calculation with the whole basis set using ghost atoms. We also considered the solvent effect using the polarizable continuum model (PCM) with water as the solvent.^50^ Therefore, we employed the CP method revised for the continuum solvent models by Gamboa-Carballo and co-workers in which first, the interaction energy in the solvent phase was calculated using supramolecular approach, and then the calculated BSSE in the gas phase was added as the correction.^51^

Single point calculations for population analysis to derive Merz–Singh–Kollman (MK) were conducted to calculate partial charges, as well as the molecular electrostatic potential (ESP).^52,53^ All calculations were performed with the Gaussian 16, Revision A.03.^54^ The nomenclature used for the structures in this study is as follows: “Li” stands for lidocaine, and “Pe” stands for pectin structures. “H” indicates the hydrogen bond interaction and “Pi” π – π interaction.

### 2.2 Experimental Studies

#### 2.2.1 Materials

Low methoxy (LM) amide pectin was provided from Herbstreith & Fox KG, Germany. Lidocaine HCl was provided by the Osel Drug Industry and Trade. Inc. TRIS (2-amino-2-(hydroxymethyl) propane-1,3-diol) and CaCl_2_·H_2_O were supplied from Sigma-Aldrich (Düsseldorf, Germany). All chemicals were of analytical grade and were not further purified. Double-deionized water was used throughout the experiments.

#### 2.2.2 Preparation of Injectable Hydrogels

Injectable hydrogels with a viscous consistency were produced by mixing different concentrations of pectin, drug lidocaine, and calcium chloride solutions, which act as a cross-linking agent. Comprehensive details of each formulation are systematically presented in Table 1.

**Table 1:** Cumulative Drug Content within the Hydrogel Resulting from the Combined DLP and D1 Solutions

	Drug loaded pectin solution (DLP)					
Code	Pectin concentration of P (w/w%)	Drug concentration of D1 (mg/mL)	Drug concentration of D2 (mg/mL)	Component ratio by volume (DLP:CLS^a^:D1)	Initial total drug amount^b^ (mg/g-hydrogel)	Cumulative drug release (mg drug/g-hydrogel)^c^
6P	6	500	-	2:2:0.68	72.3	30.2 ± 2
5P	5	500	-	2:2:0.68	72.3	45 ± 6.3
4P	4	500	-	2:2:0.68	72.3	48.4 ± 7.8
4P-1	4	500	200	2:2:0.83	169.0	60.9 ± 21.4
4P-2	4	500	200	2:2:1	180.0	77.5 ± 13.2

a CLS: Cross-linking solution (1% pectin solution and dilute CaCl_2_ solution).

b Total drug amount in hydrogel.

c Release into the isotonic solution at the end of the 30th minute.

The hydrogel formulations were created by preparing (i) pectin solution (P) in three distinct concentrations of (4%, 5%, or 6% (w/w)), (ii) drug solutions at concentrations of 500 mg/mL (D1) and 200 mg/mL (D2), each dissolved in a pH 9.1 environment; and (iii) the cross-linking solution (CLS) which is a dilute CaCl_2_ solution, augmented with 1% pectin for structural integrity.

To create the hydrogels coded as 6P, 5P, and 4P, we initiated the process by blending P with CLS for 5 min using a glass stirrer. Subsequently, the D1 solution was incorporated into this mixture, followed by an additional 5 min of stirring to ensure homogeneity.

On the other hand, to prepare 4P-1 and 4P-2 hydrogels first, a certain amount of powdered pectin was added to the D2 solution to create a drug-loaded pectin (4% w/w) (DLP) solution. After achieving complete dissolution of the pectin in the D2 solution, we introduced the CLS into the DLP solution and stirred it for 5 min. Finally, we added the D1 solution to this mixture and stirred it for another 5 min utilizing a glass stirrer. The resultant drug-loaded hydrogels, characterized by their optimized rheological properties and drug release profiles, were then carefully stored at +4 °C in their injectable form, ready for application in clinical settings.

#### 2.2.3 Structural and Morphological Characterization

The Fourier Transform Infrared Spectroscopy (FT-IR) analysis of the hydrogel was performed after it was dried under 0.2 bar of absolute pressure for 24 h at 40 °C. The analysis was conducted at room temperature using the PerkinElmer Spectrum One FT-IR spectrometer (Perkin–Elmer Inc., Beaconsfield, United Kingdom) and the total reflection (ATR) technique in the 4000–600 1/cm range. A KBr pellet was prepared for the analysis of powder lidocaine.

The surface morphology of the dried hydrogel was thoroughly investigated using Scanning Electron Microscopy (SEM) (JSM-6480LV; Jeol, Tokyo, Japan). The SEM analysis followed the same drying protocol as the FT-IR analysis, where the hydrogel was subjected to drying under an absolute pressure of 0.2 bar at 40 °C for a duration of 24 h.

#### 2.2.4 Rheological Characterization

The rheological characterization of the hydrogel was carried out Anton Paar Physica MCR 30 rheometer (Anton Paar, Graz, Austria) equipped with a plate temperature-controlled base (Viscotherm VT2) and a hood, along with CP25 (25 mm diameter cone–plate geometry) or PP25 (25 mm diameter parallel-plate geometry) measuring plates. All measurements were conducted at a stable temperature of 37 °C, and a solvent trap was used during measurements to prevent evaporation from the samples. The dynamic rheological evaluation included time sweep, amplitude sweep, frequency sweep, thixotropy, creep, and tack analysis, with all results graphically represented based on experimental data. Each measurement was replicated at least three times for accuracy.

##### Time Sweep Analysis

To perform time-sweep analysis, the storage and loss moduli of the hydrogel were measured for 45 min under a 50% strain, using a CP25 geometry. For this purpose, the drug-loaded pectin, cross-linking, and drug solutions were simultaneously placed in the measurement area of the rheometer from different directions to determine the gelation time and behavior of the hydrogel.

##### Amplitude Sweep Analysis

Amplitude sweep analyses were carried out in the amplitude range of 0.01–1000%, utilizing a PP25 geometry. These tests, performed at a constant frequency of 6 Hz, aimed to accurately determine the Linear Viscoelastic (LVE) region. Additionally, they facilitated the identification of specific strain or stress values corresponding to the flow point.

##### Frequency Sweep Analysis

In the oscillation frequency sweep analysis, the PP25 geometry was utilized. Measurements were conducted maintaining a gap distance of 1.2 mm. Both the storage modulus (*G*′) and loss modulus (*G*″) were quantified through a frequency sweep ranging from 0.1 to 100 rad/s within the Linear Viscoelastic (LVE) region.

##### Thixotropic Oscillatory Strain Sweep Analysis

The thixotropic oscillation strain test was performed with a frequency of 6 Hz to evaluate the capacity of the sample for syringe delivery and rapid self-healing. Initially, a high strain magnitude of 1000% was applied for 30 s in the first stage of the tricycle test, followed by a low strain (0.2%) step at the end of each cycle. The storage modulus ratio after and before the cycle is a quantitative measure of the recovery rate (RS) of the hydrogel at the end of the third cycle of high stress (eq 4).
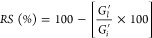
4*G′*_*i*_ and *G′*_*l*_ are the average storage modulus of the initial (Region I) and third cycle, respectively.

##### Creep-Recovery Analysis

To conduct the creep-recovery tests, a shear stress of 10 Pa was applied for a duration of 300 s, which is referred to as the creep phase. After the creep phase, the hydrogels were allowed to recover their strain for an additional 600 s, which is called the recovery phase. The strain and creep compliance of the hydrogels were recorded as a function of time during both the creep and recovery phases.

##### Tack Analysis

Adhesion (Tack) measurement was carried out using the CP25 geometry. The measurement tip was pulled at a speed of 5 mm/s. The sample was subjected to a 1000 1/s shear rate before the measurement tip was pulled, and the change in normal force with opening and time was studied. Adhesion energies were obtained by calculating the areas of the opening-force and time-force curves.

During the adhesion analysis, a load was applied to the solution by the measurement tip of the rheometer. The measurement tip was then retracted at a constant speed, and the force applied by the sample to the measurement tip was measured as a function of time and opening. The force reaches a maximum point and then begins to decrease; this stage is the relaxation phase. The stage where the force decreases to a constant zero value is the detachment stage. eq 5 was used to calculate the adhesion energy from the detachment force.
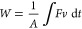
5

Here, *W* represents the adhesion energy, *A* represents the area under the curve, *F* represents the force, *v* represents the detachment speed, and *t* represents the time. Since the speed and area of the measurement tip were constant during the experimental period, the adhesion energies of the samples were able to be compared to the area of the force curves against time.^55^

In addition to the force-gap and force-time values, the tack test integral (N.s) was able to be used to quantify the tackiness of the material. The tack test integral represents the total energy absorbed by the material during the test and provides a measure of its tackiness. It can also be expressed in units of joules (*J*), which is the energy absorbed per unit of time during the test.^56,57^

#### 2.2.5 Drug Release Behavior

The *in vitro* drug release of the pectin hydrogels was carried out in pH 7.4 PBS buffer, isotonic, and pH 5.0 citrate buffer solutions environments. For the drug release measurements, 1 g of the drug-loaded pectin hydrogel was placed in amber vials, and 50 mL of buffer solution was added. The vials were then subjected to uniform mixing using an orbital mixer, maintained at a controlled temperature of 37 ± 1 °C throughout the analysis period. The concentration of lidocaine in the solution was monitored for 30 min, which was the surgical operation time, utilizing a LAMBDA 1050 UV spectrophotometer (PerkinElmer Corp, Waltham, MA, USA) at 265 nm.^58,59^ During the process, solution samples were periodically extracted using a 900 μL micropipette at predetermined intervals. Post absorbance measurement, these samples were promptly returned to their respective vials to maintain solution consistency. The drug concentration was calculated employing preprepared calibration curves based on the Lambert–Beers law. The experiments were repeated at least three times under the same conditions.

### 2.3 *In Vivo* Experiments

In the study, 46 male Sprague–Dawley rats that were 10 weeks old and weighed ∼ 300 ± 20 g were used in compliance with the regulations of the institutional ethics committee. The animals were randomly divided into two main groups: the experimental group (n = 22), where microvascular anastomosis was performed in the produced hydrogel medium, and the control group (n = 20), where only conventional microsutures were used for anastomosis. The rats were kept under a 12/12 h light/dark cycle and given food and water ad libitum.

**Figure 1:**
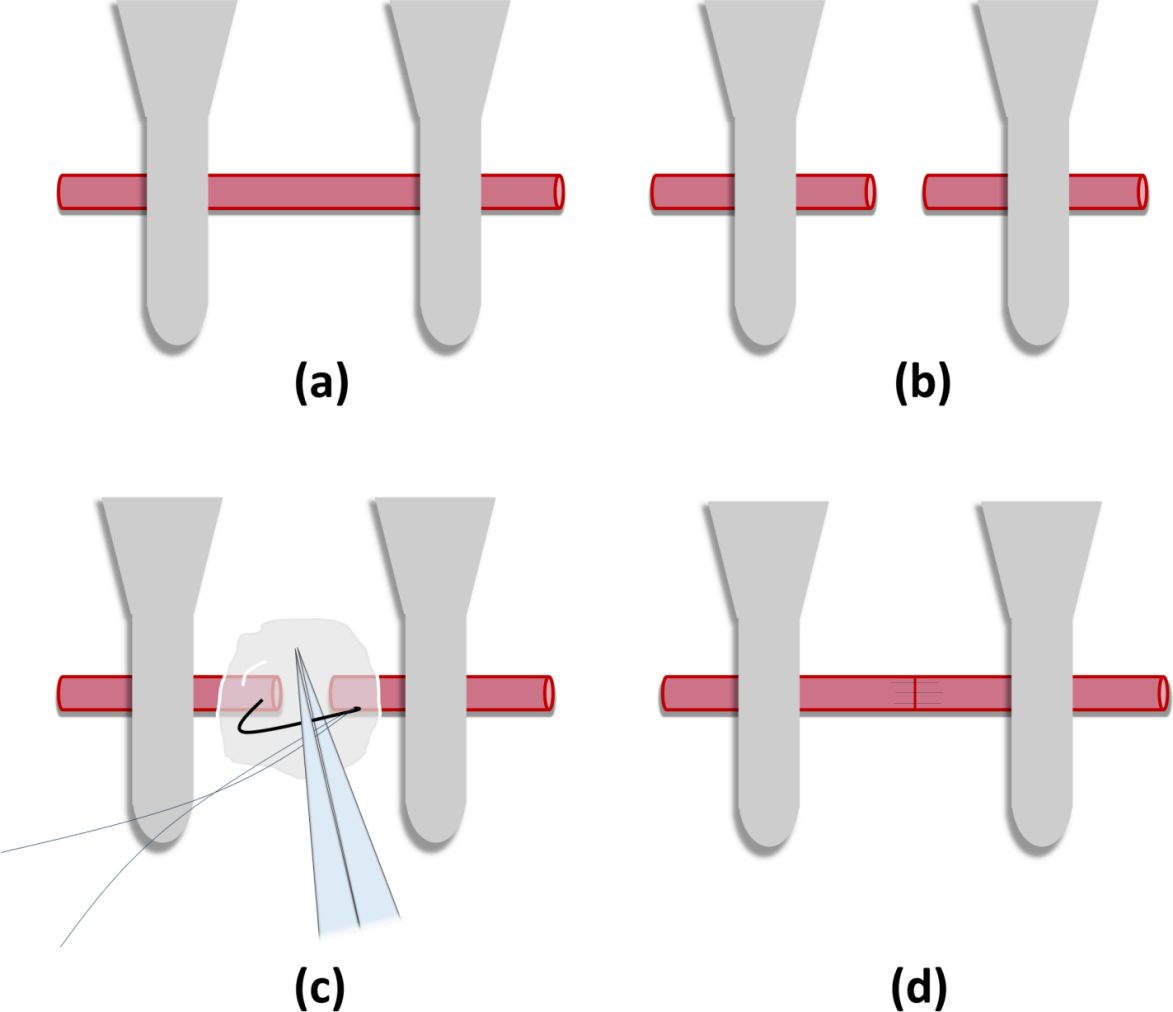
(a) An approximator was placed either on the artery at 1.5 cm from each other under the surgical microscope (× 16 magnification), (b) Arteriotomy was performed, (c) In the experimental group, the pectin-based hydrogel was injected intraluminally and into the anastomosis site with a 26-gauge injector. Stent effect of the hydrogel preventing the collapse of the lumina of the vessel ends can be observed, and (d) Anastomosis was completed.

Microvascular anastomosis time and early patency rate: The rats were anesthetized using a mixture of 30–35 mg/kg ketamine and 10 mg/kg xylazine injected intraperitoneally. In accordance with the rules of local asepsis, the inguinal area, which was the surgical area, was shaved and cleaned with povidone-iodine. According to the femoral artery and vein end-to-end anastomosis model, an incision was made over the inguinal fold, and the inguinal fat pad was raised and preserved as a flap over the pedicle. Inguinal ligament was found and immediately distal to it, the femoral neurovascular bundle was exposed. The lower abdominal muscles were retracted medially, and the dissection was continued on the femoral pedicle under the microscope at ×10 magnification until distal bifurcation. Branches of the femoral vein and then the femoral artery (Figure 1a) were identified and carefully ligated with 9.0 Polyamide sutures. After the vascular adventitia was dissected and removed from the periphery of the vessel, the diameters of the arteries and veins were measured before anastomosis with a microsurgical crack width ruler under the microscope.

After the ruler was removed, two microvessel clamps were placed either on the artery or vein at 1.5 cm from each other (Figure 1a). Under the surgical microscope, first, the vessel was cut to full-thickness (Figure 1b), and the ends were exposed. In the experimental part, the pectin-based hydrogel was injected intraluminally (Figure 1c) and into the anastomosis site with a 26-gauge injector. The onset times of anastomoses were recorded separately for arteries and veins, and they were performed using 8/0, 9.0, or 10/0 Polyamide nonabsorbable standard microsurgery interrupted sutures at × 16 magnification (Figures 1d). In the experimental group, before the last stitches of the artery and vein anastomoses, the gel in the intravascular and surgical environment was washed with saline. Distal and proximal clamps were opened in that order, and arterial and vein anastomosis times were recorded in all rats. Anastomosis time was defined to begin with the moment of arteriotomy or venotomy and end with the removal of clamps. In cases of leakage, the clamps were reapplied, and additional sutures were placed in the leaky openings. In such cases, this extra duration was added to the anastomosis time. After the anastomoses were completed, the inguinal fat pad flap prepared during dissection was transposed on the pedicle. After 30 min, the patency of the artery and vein was examined under × 16 magnification.

#### Assessment of Lidocaine Release

: To demonstrate the efficiency of lidocaine release from the pectin-based hydrogel, the diameters of both femoral artery and femoral vein were measured before and after anastomosis in the gel (n = 22) and control groups (n = 20) using a metric ruler. After the initial surgery, animals in the gel and control groups were randomly divided into first week, sixth week, and sixth month groups.

Second, we chose to use an *in vivo* model to measure the response of vessels in vasospasm after dissection to most closely simulate a real clinical scenario.^60,61^ After pathological samples were obtained from the right-sided femoral vessels, the left-sided femoral pedicle was exposed surgically. Exposure of the femoral artery was achieved by opening the perivascular sheath under microscopic magnification and separating the left-sided femoral artery from the femoral vein and femoral nerve in the area between the inguinal ligament and the origin of the epigastric branches, and the Murphy branch of the artery was ligated. Continuous saline irrigation was performed to prevent desiccation during surgery. The diameter of the left femoral artery was measured with a crack-width ruler just before the gel was applied under the microscope with ×16 magnification. Femoral artery diameters were recorded at fifth, tenth and 15th minutes of gel application. Digital video-records were obtained during the whole process (Video S1).

#### Vascular Imaging with Doppler Ultrasound and CT Angiography

: In the sixth week (n = 13) and sixth month (n = 15) rat groups, the bilateral femoral artery diameter, flow velocity, patency, femoral vein diameter, and patency were evaluated with a 6–12 mHz linear probe Doppler Ultrasound (Ge Logic). The femoral artery flow rate was calculated according to eq 6(62,63) after measuring the femoral artery diameter and Peak Systolic Velocity (PSV) by Doppler ultrasound and was recorded in mL/s:
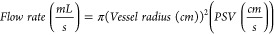
6

To compare the gel and control groups, normal values were obtained from the left femoral artery and vein of the same animals that had not undergone surgery. After Doppler measurements were performed in all groups, computerized tomography angiography (Philips Allura Clarity) was performed by cannulizing the aorta. Then, after administering 0.1–0.2 mL of Ultravist contrast agent at a dose of 300 mg/mL with the help of a catheter through the aorta, femoral artery images were taken and recorded (Video S2). After the radiological evaluation was completed, femoral artery and vein samples were obtained for histopathological examination, followed by euthanasia.

#### Histological and Immunohistochemical Analysis

: At the end of the first week, sixth week, and sixth month, 2 cm-long femoral artery and vein samples harvested from the rats in the experimental and control groups were fixed in a solution containing 10% formaldehyde for 24 h. Left-sided femoral arteries and veins of the animals belonging to the first week group were also harvested as control. All materials were evaluated histologically and immunohistochemically. After blocking, consecutive sections of 4 μm thickness were taken for routine H&E, Verhoeff van-Gieson (EVG), periodic acid-Schiff (PAS), Reticulin, CD31 and CD68 (Abcam) staining, respectively. The number and types of inflammatory cells, the number of histocytes and giant cells, and the amount of fibrosis were evaluated semiquantitatively by comparing the intact area/anastomosis area. Endothelial integrity was assessed using CD31 by immunohistochemical method, and histiocyte count was performed on slides stained with CD68. Two different pathologists made all measurements separately. Values were recorded by taking the average of two measurements.

##### 2.3.1 Statistical Analysis

The DATAtab Online Statistics Calculator (Austria, 2022) was used for statistical analysis. Kolmogorov–Smirnov and Shapiro-Wilk tests were used as initial analyses to evaluate the normal distribution. For comparisons between two groups, Mann–Whitney U, Fisher’s exact test, and chi-square tests on slope were used for nonparametric data. Analysis of Variance (ANOVA) test was used for comparisons of more than two groups of parametric data. The results were evaluated in the 95% confidence interval and *p* < 0.05 was defined as statistical significance.

## 3 Results and Discussion

### 3.1 Molecular Dynamics and Quantum Mechanics Calculations

One of the aims of the injectable pectin hydrogel is to deliver the vasodilator lidocaine^64−66^ while enabling the supermicro-vascular anastomoses by preventing the collapse of arteries or veins. The drug release behavior of the hydrogel is expected to depend on the molecular interactions between both lidocaine-pectin hydrogel and lidocaine-lidocaine molecules. The preparation of the drug-loaded injectable pectin hydrogel involves blending pectin, lidocaine, and cross-linker solutions at different pH values, as will be detailed in the next section. Therefore, computational studies were performed to investigate possible interaction models of lidocaine-lidocaine and lidocaine-pectin matrix at different pH values to calculate intramolecular and intermolecular interaction energies that can give useful insights about the suitability of loading/releasing lidocaine on/from the pectin hydrogel.

Lidocaine is a synthetic aminoethyl amide used as a local anesthetic and has a p*K*_a_ of 7.7.^42^ At lower pH values, lidocaine molecules are protonated thus increasing their hydrogen bond interaction capability. pH values of the drug solutions (D1 and D2 in Table 1) in the experimental studies were determined as 9.1, where lidocaine molecules are in their neutral form. However, the pH value decreased to 4.5 with the addition of pectin (P in Table 1) to the solution, where lidocaine molecules are expected to be protonated. When protonated, lidocaine is likely to increase its ability to make hydrogen bond interactions. However, since molecules undergo conformational changes, whether this increased ability will improve their interactions with other components may vary from system to system. Molecular dynamics (MD) simulations considered both pH conditions to monitor the type and extent of molecular interactions in the systems. Before the analysis of the trajectories, MD simulations were controlled if they reached equilibrium in terms of energy (Figure S1). Based on the radial distribution function analysis (Figure S2), neither neutral nor protonated lidocaine molecules interacted with Ca^2+^ ions of CaCl_2_, which acts as a cross-linker to coordinate – COOH/-COO^–^ groups of the pectin chains to form the hydrogel. In order to obtain a better description of the electrostatic interactions and interaction energies, quantum mechanical calculations at DFT level of theory were carried out for the molecular complexes. The input structures for DFT calculations were chosen from MD simulations to include several conformations especially bearing the favorable π–π and hydrogen bonding interactions (Figure S3). The calculated interaction energies for the selected neutral and protonated lidocaine-lidocaine systems are given in Table 2.

**Table 2:** Neutral/Protonated Lidocaine-Lidocaine and Protonated Lidocaine-Deprotonated Pectin Interaction Energies

Model Structure	Interaction energy (kcal/mol)
LiLi-HI	–9.8
LiLi -HII	–15.4
LiLi -HIII	–8.8
LiLi-PiI	–13.6
LiLi -PiII	–10.0
LiLi -PiIII	–14.4
LiLi-H-Pi	–11.5
Li^+^Li^+^-HI	–17.4
Li^+^Li^+^-HII	–14.5
Li^+^Li^+^-HIII	–17.6
Li^+^Li^+^-PiI	–9.7
Li^+^Li^+^-PiII	–8.9
Li^+^Li^+^-PiIII	–11.7
Pe^–^Li^+^-I	–17.5
Pe^–^Li^+^-II	–9.8
Pe^2–^Li^+^-III	–17.9

Neutral lidocaine molecules make both hydrogen bonding and π–π interactions, with interaction energies up to −15.4 kcal/mol and −14.4 kcal/mol, respectively (Figure S4a). Among the protonated lidocaine-lidocaine conformers, the most stable complex is the Li^+^Li^+^-HIII having a hydrogen bonding interaction (Figure S4b) with a distance of 1.80 Å and an angle of 171.6° indicating a strong interaction between them. The intermolecular stabilizing interactions are stronger for hydrogen-bonded structures than π–π stacking ones although the most stable complex with a π – π stacking interaction, i.e. Li^+^Li^+^-PiIII, has an intramolecular hydrogen bonding in one of the complexes that can contribute to stabilization (Figure S4b). With the protonation, the strength of π – π stacking interactions was decreased whereas the hydrogen bond interactions became dominant.

When combined with the pectin solution, the pH decreases leading to protonation of the lidocaine molecules throughout the stirring process. Protonated lidocaine molecules are expected to either interact with the pectin or themselves. If they prefer to interact with the pectin, three possible interacting sites are plausible at low pH; either with the carboxylate or carboxylic acid group, or hydroxyl groups of the galacturonic acid unit (Figure S5a). MD simulations conducted in explicit solvent did not indicate an interaction of the protonated amine with the hydroxyl groups. The interactions were especially concentrated with the deprotonated carboxylate groups (Figure S5b). In quantum mechanical calculations, the pectin oligomers were shortened to three units to enable the calculations at a high level of theory. For the pectin-lidocaine structures selected from MD trajectories, the pectin oligomers either have −1 or −2 charges (Figure S5a). The calculated interaction energies between the pectin and lidocaine molecules are given in Table 2. The most stable pectin-lidocaine complex structures are shown in Figure S6a. When the ESP charge distribution maps are examined (Figure S6b), the positive charge is dominant on the surface of the most stable lidocaine dimer (Li^+^-Li^+^-HIII), the negative charge is concentrated on the carboxyl group of the deprotonated alpha-D-galacturonic acid unit for the Pe^2–^-Li^+^-III which provides a surface for interaction with another lidocaine molecule. The findings thus suggested that lidocaine molecules are able to interact with each other and the pectin chains, especially through hydrogen-bond interactions.

The computational study proposed that with a decrease in pH, the ability of lidocaine molecules to make π – π interactions decreases, which would prevent the stacking of the drug molecules. This facilitates hydrogen bonding interactions of lidocaine both with pectin and themselves with a comparable interaction energy of ∼ −17 kcal/mol. The presence of hydrogen bond interactions between lidocaine and pectin implied that lidocaine is likely to be slowly released from the pectin if a low drug concentration is used, as was previously shown for procaine-pectin systems.^28^ Similarly, a high concentration of pectin would create a highly cross-linked hydrogel, increasing the diffusion path of the lidocaine molecules and resulting in a low amount of drug release.^26^ The simulations also showed that lidocaine molecules did not have notable interactions with Ca^2+^ ions, implying that Ca^2+^ ions in the solution would be available to cross-link pectin chains. These results suggested that lidocaine can be used in the injectable pectin hydrogel system with drug delivery.

### 3.2 Hydrogel Preparation

Low methoxy pectin hydrogels were prepared by employing the “ionotropic gelation method”, which involves cross-linking of Ca^2+^ ions with ionized carboxyl groups (−COO^–^) in pectin chains.^26−29,31,32^ By mixing different concentrations of lidocaine-containing pectin solution with calcium chloride solution, pectin hydrogel with a viscous consistency was produced (6P, 5P, and 4P hydrogels). A noted challenge in this process is the rapid gelation between the pectin chains and Ca^2+^ ions, often leading to the formation of a hard, outer shell on the hydrogel. This shell can impede the ease of administration via syringe, hinder the maneuverability of surgical instruments and sutures within the hydrogel matrix, and potentially cause issues with optical clarity.^67−69^ To address this, a novel cross-linking strategy was developed. In this approach, the CaCl_2_ solution is encapsulated within a dilute low methoxy pectin solution, forming what is referred to as the cross-linking solution (CLS). In this way, the interaction rate between the Ca^2+^ ions and COO^–^ on the pectin chains is slowed down, preventing the formation of an undesirable hard shell on the outer surface of the hydrogel, and also the possibility of low-viscosity CaCl_2_ solution flowing and interacting with the tissues is eliminated. Thus, transparent hydrogels coded 4P-1 and 4P-2 that allowed a high degree of light transmission, enabled a clear vision during suture placement, and released lidocaine during operation, were developed with only pectin, Ca^2+^ ions, and drug molecules, without the need for additional materials.

The developed hydrogel was a fast-gelling and injectable formulation that could be easily administered using a 21G syringe before the operation. In the literature, there is a peptide-based injectable polymer synthesized for anastomosis applications by Smith et al. that can undergo sequential sol–gel and gel–sol phase transitions.^16^ However, this peptide-based material lacks drug-carrying capabilities and relies on light radiation for degradation and environmental removal postanastomosis. In contrast, the poloxamer-based gel developed by Chang et al. is constrained by its temperature-dependent sol–gel transitions, which diminishes its practical utility.^7^ These transitions pose a significant limitation, especially in the context of surgical environments.

The hydrogel developed in our study, however, not only facilitates the release of lidocaine but also exhibits spontaneous self-degradation following the completion of the anastomosis process.^7^ A key advantage of this hydrogel is its relatively stable viscosity under standard operating room conditions. This stability is particularly beneficial when contrasted with poloxamer hydrogels, which undergo a solid-gel transition at temperatures below the human body temperature, specifically at 25 °C. Such temperature sensitivity can lead to solid–liquid behavior transitions during surgeries, potentially resulting in perioperative complications, including embolism. Thus, our hydrogel offers a more reliable and safer alternative for surgical applications.

### 3.3 Lidocaine Release Behavior of the Hydrogels

Given that the success of surgical procedures involving vascular anastomosis critically depends on the anastomosis to remain open, a critical requirement for the material used in such operations is to release a specific amount of lidocaine molecules. This release should start with an initial burst, continuing over a period of 30 min to prevent vasospasm. Therefore, in this study, our primary focus was on meticulously evaluating the lidocaine release dynamics of the prepared hydrogel formulations. Subsequently, we characterized the most suitable sample based on their exhibited drug release performance.

**Figure 2:**
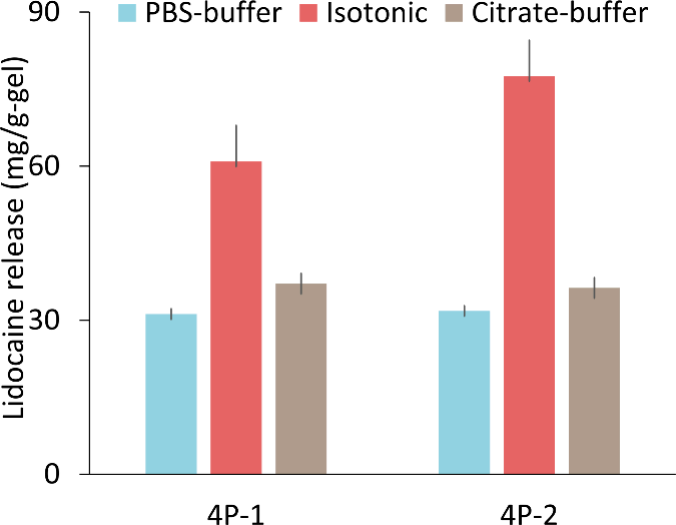
Lidocaine release from 4P-1 and 4P-2 hydrogel in PBS (pH 7.4), isotonic (pH 5.8) and citrate buffer (5.0) environments.

Drug release studies were performed on hydrogel formulations with varying initial pectin concentrations (4%, 5%, and 6%) in a 37 °C isotonic environment for 30 min which is the estimated duration of anastomosis operation. Table 1, Figure 2, and Figure S9 present the results. To achieve a high cumulative drug release with a burst release during rapid anastomosis surgery, a cross-linking solution was added to the pectin matrix before the addition of the drug solution. This approach allowed Ca^2+^ ions in the formulation to interact with the charged groups on the pectin chains, reducing the likelihood of added drug molecules interacting with the chain atoms through hydrogen bonding interactions that were indicated by the computational findings. Therefore, this method effectively achieves the desired burst release during the operation. The studies revealed a negative correlation between the initial pectin concentration and drug release. Cumulative drug release for 6% (6P), 5% (5P), and 4% (4P) (w/w) formulations was measured to be 30.2 ± 2.0, 45 ± 6.3, and 48.4 ± 7.8 mg lidocaine/g-hydrogel, respectively (Table 1). It was observed that an increase in the concentration of pectin within the solution led to a reduction in cumulative drug release. This effect is attributed to the narrowing and elongation of the diffusion path caused by chain entanglement,^26^ for the lidocaine molecules having an affinity for the pectin chains shown by the computational results.

To increase cumulative lidocaine release while maintaining a specific viscosity level required for the operation, we dissolved powder pectin (4% w/w) in a lidocaine solution (200 mg/mL). After adding the cross-linking solution, we added an external drug solution of 500 mg/mL to the matrix in two different formulations (4P-1 and 4P-2) (Table 1). As the amount of the external drug solution was increased in the hydrogel formulation (4P-2), we observed an increased drug release of 77.5 ± 13.2 mg lidocaine/g-hydrogel compared to the 4P-1 formulation. Afterward, drug release experiments were conducted for 30 min under two additional conditions: pH 7.4 PBS and pH 5.0 sodium citrate environments. These conditions were selected to mimic the operating conditions for the 4P-1 and 4P-2 formulations, which demonstrated a higher drug release in comparison to the other formulations in the isotonic environment, as shown in Table 1 and Figure 2.

After a thorough evaluation of all experimental results, a decision was made to focus on an in-depth study of the 4P-2 hydrogel. This hydrogel was identified as achieving the most desirable and highest drug release characterized by a high initial burst release (Figure S9). This release pattern is intended to provide an immediate therapeutic effect during the early stages of the anastomosis operation in an isotonic environment. The proposed detailed study will encompass structural, morphological, and rheological characterization of the 4P-2 hydrogel, to further understand and optimize its properties for surgical applications.

### 3.4 Structural, Morphological, and Rheological Characterizations of the Hydrogel

As explained in Supporting Information, the FT-IR spectra of the hydrogel indicated that the slight shifts in the characteristic peaks of pectin and lidocaine suggested the successful incorporation of lidocaine molecules into the polymer matrix (Figure S7). These results suggest that no undesired new structures were formed during the synthesis of hydrogels, and the drug molecules were successfully dispersed into the hydrogels without disrupting the molecular integrity. Furthermore, the SEM images given in Figure S8 revealed that the 4P-2 hydrogel formulation produced a porous matrix that is suitable for drug release.

Rheological analysis, a critical aspect in the study of flow and deformation characteristics of materials, holds significant importance in biomedical applications, particularly for injectable gels. This analysis is pivotal in understanding the viscoelastic properties of such materials, which directly influence their performance in medical applications.

We first determined the gelation point and initial stability of the hydrogel 4P-2 through a comprehensive time sweep analysis. Subsequently, we conducted an extensive investigation of its rheological properties, across five primary categories to ensure its suitability for use in anastomosis operations; (i) injectability was assessed through strain sweep analysis, (ii) stability, syringe delivery, self-healing properties, and poststability were evaluated via a 3-region connected rheological analysis, which comprised two individual time sweep tests and one 3-cycled thixotropic oscillatory strain amplitude sweep measurements, (iii) spontaneous degradability of the hydrogel at the end of the operation were tested by a 3-region 30 min time sweep analysis and frequency sweep analysis, (iv) durability and reliability were examined by creep-recovery analysis, and (v) low adhesion to surgical equipment was confirmed using tack analysis.

#### 3.4.1 Gelation Point

Rapid cross-linking between pectin chains and Ca^2+^ ions can lead to the development of a hard outer shell on the surface of hydrogels. Such an outcome poses significant challenges in surgical contexts, potentially hindering the functionality and applicability of the hydrogel. To address this issue, the gelation rate of the 4P-2 hydrogel was intentionally slowed down by entrapping the CaCl_2_ solution within a dilute pectin solution.

As the lidocaine-loaded pectin solution begins to gel, a cross-linked network forms, causing both *G*′ and *G*″ to increase as shown in Figure S10. Notably, the rate of increase of *G*′ is higher than that of *G*″ indicating that the elastic properties of the gelling hydrogel are becoming predominant.^70^ Consequently, a crossover point is reached where *G*′ exceeds *G*″. The time required to reach this crossover point is often referred to as the gelation time for the solution.^71^

The gelation time of 4P-2 hydrogel was determined to be approximately 22.8 min (Figure S10). Furthermore, *G*′ of 4P-2 hydrogel increased over time starting at 2.5 Pa and reaching 45.6 Pa by the 45th minute, indicating an increase in cross-linking density and consequently greater rigidity.

The phase angle (tan δ = *G*″/*G*′) provides deeper insight into this phenomenon. A decrease in tan δ typically indicates an increase in structural rigidity.^32,72^ According to our data, just after the gelation point where tan δ equals 1, the tan δ of the hydrogel decreased to 0.348 by the 45th minute (Figure S11) as the cross-linking density increased over time. This increase in cross-linking density made the gel more solid-like, enhancing its elasticity and stability.^73^ These findings demonstrate the stability of the 4P-2 formulation and its suitability for use in anastomosis operations.

#### 3.4.2 Injectability, Stability, Self-Healing, and Spontaneous Degradation Properties of the Hydrogel

The flow point, commonly identified as the crossover point where the storage modulus (*G*′) is equivalent to the loss modulus (*G*″), is one of the characteristics for evaluating the injectability of hydrogels. In our study, the flow point of 4P-2 was observed when the storage modulus (*G*′) reached 19.54 Pa, coupled with a flow point strain (γ_F) of 215% (Figure S13c, d). This relatively low flow point signifies a highly flexible structure, which is vitally important for its applicability in surgical contexts.

In comparison, as detailed in the literature, this flow point is lower than that of some other polysaccharide hydrogels, such as the hydrogel made from oxidized pectin and adipodihydrazide-functionalized pectin exhibits a flow point strain of approximately 400%.^74^ Similarly, injectable hydrogels composed of pectin aldehyde and poly(N-isopropylacrylamide) display flow point strains of 170%, 290%, and 530% varying with the concentration of the cross-linker.^33^ Additionally, the supramolecular hydrogel based on hyaluronic acid and beta-cyclodextrin typically shows a flow point strain of around 250%.^75^ These comparative analyses underscore the 4P-2 hydrogel’s lower resistance to flow and its enhanced injectability, particularly when administered through a 21G syringe, as demonstrated in Figure S13.

**Figure 3:**
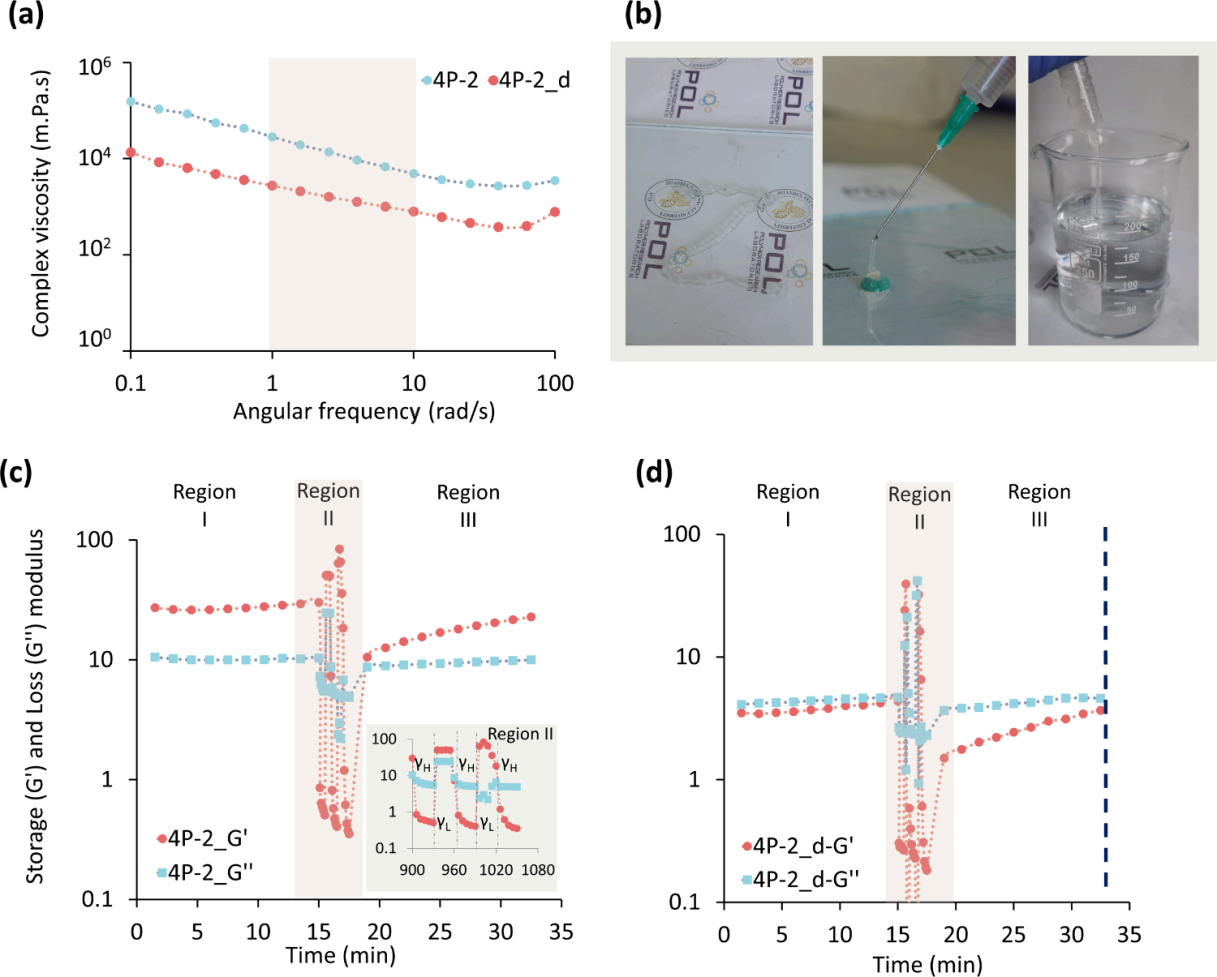
Rheological characterization of the hydrogel. (a) complex viscosity as a function of the angular frequency of 4P-2 and 4P-2_d hydrogels, demonstrating shear thinning ability and significantly decreasing the viscosity of swelled at 37 °C hydrogel which is coded 4P-2_d, when compared to unswelled hydrogel 4P-2, (b) photographs of the hydrogel showing that it can be injected continuously with a 21 G for writing smooth letter ‘Z’, (c) rheological assessment showing the stability (Region I), shear thin/recovery (Recovery II), and poststability (Recovery III), with an inset magnifying Region II to emphasize the hydrogel’s gel–sol transition under a high shear strain of 1000% γ_H and sol–gel transition under a low shear strain of 0.2% γ_S, and (d) time sweep analysis for 4P-2_d hydrogel, which mimics the condition of the hydrogel at the end of the surgical operation. The logo used as a photo background in Figure 3b belongs to the ITU Polymer Research Group and has been used with the permission of the group leader.

Moreover, frequency sweep analysis demonstrated that the hydrogel exhibits shear thinning behavior (Figure 3a). In line with findings in the literature, the complex viscosity decreases linearly with increasing frequency, which allows the hydrogel to flow more easily through the syringe and ensures accurate dispensing as illustrated in Figure 3a.^76−78^ The results demonstrate a linear decrease in complex viscosity corresponding to the frequency on a double logarithmic scale, marked by a sharp slope (0.63), indicative of a pronounced shear-thinning behavior in the formulations. The shear thinning property implies that the hydrogel formulation can dynamically adapt and withstand deformation. This property is due to the instant arrangement of the hydrogel network structure into layers that flow in the direction of shear.^79,80^

Macroscopic visual injectability experiments were conducted providing further evidence of the hydrogel’s injectability. These experiments demonstrate that the hydrogel can be injected continuously and that the deformed hydrogel can rearrange into a well-shaped hydrogel letter-Z after injection (Figure 3b).

To further evaluate the (i) stability, (ii) injectability and self-healing ability, (iii) post stability, and (iv) degradability of the 4P-2 hydrogel at the end of the surgical operation of the 4P-2 hydrogel, a rheological analysis comprising three regions was conducted (Figure 3c). At the beginning of the test, to assess the stability of 4P-2 hydrogel, 0.2% strain was applied for 15 min under Region I. The data indicated that *G*′ > *G*″ and remained stable throughout 15 min, demonstrating that the 4P-2 hydrogel is highly stable. Subsequently, to mimic the syringe delivery and observe the self-healing ability of the hydrogel during injection into the vessel lumen followed by its recovery into a hydrogel state, a 3-cycled thixotropic oscillatory strain amplitude sweep measurements were conducted (Region II). As clearly shown in the inset of Figure 3c, the application of high shear strain (γ_H= 1000%) resulted in a viscous-like structure (*G*′′ > *G*′), indicating gel–sol transition (a flowable structure) of the system. However, upon reducing the applied shear strain to a low value (γ_S= 0.2%), the hydrogel immediately reversed its initial *G*′, demonstrating a rapid restoration of its original structural integrity. Based on the calculations using the RS formula (eq 4), the 4P-2 hydrogel exhibits an exceptional healing capacity, fully recovering 100% of its initial *G*′ (Table S2).^72^ The rapid self-healing mechanism of this hydrogel relies on dynamic reversible both inter- and intramolecular hydrogen bonds as well as electrostatic interactions between COO^−^ groups of pectin chains and cross-linker Ca^2+^ ions. These interactions can break or reorganize when the hydrogel is deformed.^28,81,82^ Thus, we can conclude that the hydrogel exhibits the capability to effectively seal any cracks that may form during suturing or the movement of surgical equipment. At the end of the thixotropic cycle test of 4P-2 hydrogel a 15 min poststability step (Region III) was conducted at 0.2% strain (Figure 3c). The storage modulus (*G*′) of the hydrogel, which initially measured 27.2 Pa, exhibited a decrease to 22.8 Pa by the end of the testing period. The data showed that *G*′ of the 4P-2 was slightly decreased indicating an initial spontaneous decomposition.

Finally, we focused on the spontaneous breakdown of the hydrogel network, specifically the gel–sol transition, which is a desirable property for the anastomosis operation after completion. To assess the spontaneous degradation of the hydrogel, two pieces of 4P-2 hydrogels were placed in Petri dishes containing pH 7.4 TRIS buffer at 37 °C that mimic the biological operation environment. These hydrogels were labeled as 4P-2_d. While the first sample 4P-2_d underwent a 3-step 30 min time sweep analysis (Figure 3d), a frequency sweep analysis was conducted for the other sample (Figure S14). According to the time sweep results shown in Figure 3d, a significant decrease in the *G*′ values was observed in Regions I and III for the 4P-2_d hydrogel when compared to the 4P-2 sample.

In the angular frequency range of 1 to 10 rad/s, comparative analysis revealed that the average storage modulus (*G*′) of the 4P-2_d hydrogel decreased 10-fold compared to the 4P-2 hydrogel, illustrating a significant reduction in the structural integrity of the real three-dimensional network.^83^ Initially, *G*′′ of the hydrogel was 0.52 Pa; it increased to 77.8 Pa by the end of the test. Additionally at frequencies higher than 6.31 rad/s, with *G*′′ becoming higher than *G*′, the hydrogel structure transitioned to its viscous phase, indicating structural destruction (Figure S14). This behavior, indicative of the hydrogel’s decomposition, is ideal for anastomosis applications.^84^ These observations strongly indicate that the hydrogel network is prone to spontaneous degradation when subjected to the surgical environment for around 30 min, a critical insight for its application in medical procedures. Hence, eliminating the step of removing the hydrogel from the environment at the end of the anastomosis could be another potential benefit of 4P-2 hydrogel.

We also assessed the creep and recovery analysis of the tested 4P-2 formulation. The creep recovery of hydrogels to be used in an anastomosis is an important factor to consider, as it affects the stability and durability of the surgical joining of two hollow organs. The results demonstrated that the tested 4P-2 formulation was capable of enduring severe compression (Figure S15, Figure S16).

#### 3.4.3 Adhesion Performance of the Hydrogel

For anastomosis operations, it is desirable for the material to have high viscosity and minimal adhesion to surrounding tissues and surgical instruments, as well as minimal tackiness to sutures. To test the adhesive strength of a hydrogel to surgical equipment and suture, tackiness analysis was performed as described in the literature.^55,85^ The results of the tack test analysis are presented in Figure S17. Generally, a low tack test integral value (0.94 N.s) is desirable for surgical materials, as it implies low tackiness (Table S2).^56,57^ Based on this perspective, it can be stated that the synthesized hydrogel exhibits low tackiness to the surrounding tissues and surgical materials.

In conclusion, based on the above rheological and macroscopic analyses, the produced 4P-2 hydrogel exhibits; (i) a shear-thinning property, allowing for continuous injectability through a needle (Figure 3a,b, Figure S12), (ii) the required amount of lidocaine release for the entire operation period (Figure 2), (iii) self-healing ability (Figure 3c), (iv) minimal adhesion to surgical instruments (Figure S17, Table 2), and (v) spontaneous dissolution with outstanding gel–sol transition behavior when exposed to blood flow shear stress at the end of a 30 min operation (Figure 3d, Figure S14).

### 3.5 *In Vivo* Experiments

#### 3.5.1 Microvascular Anastomosis Time and Early Patency Rate

**Figure 4:**
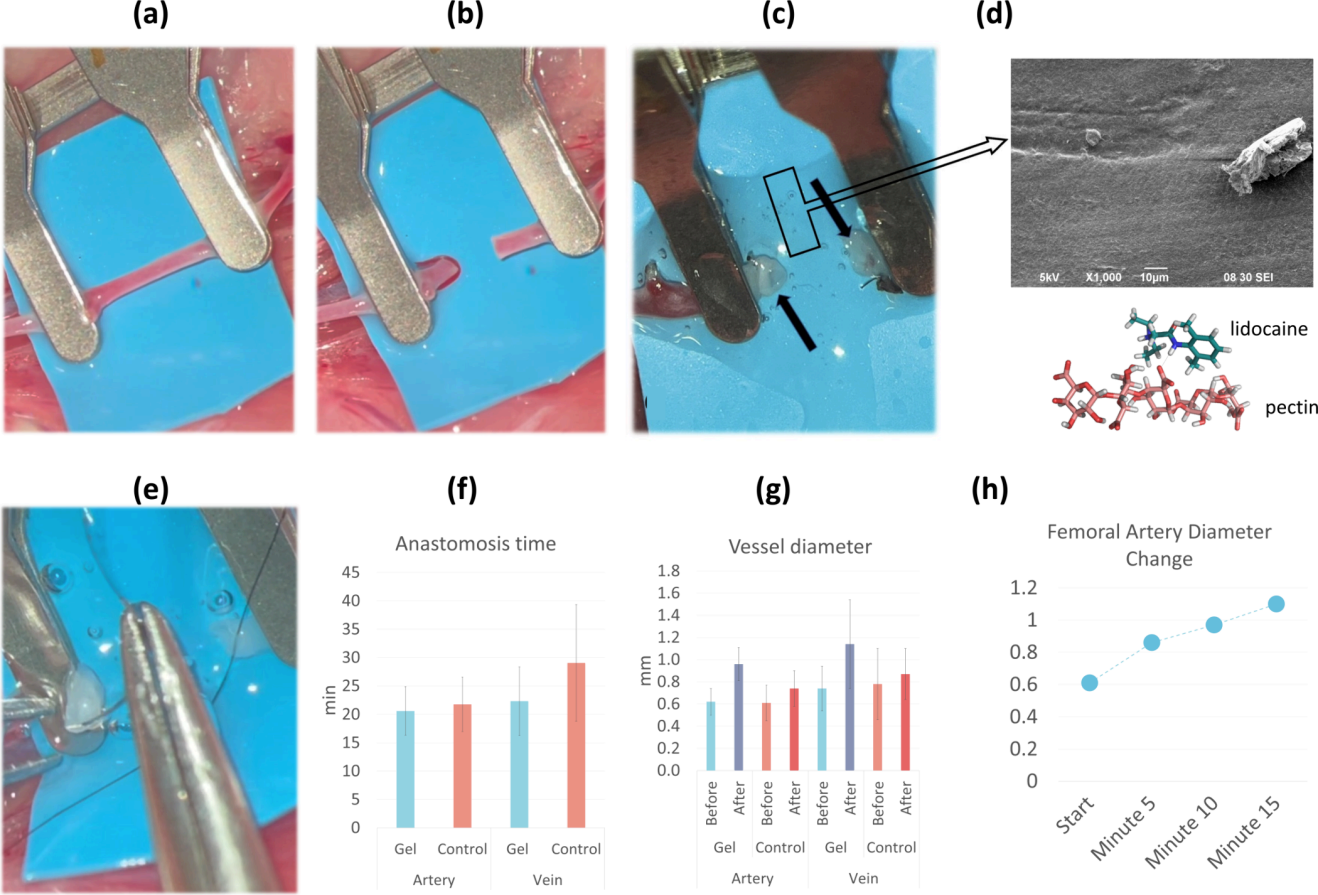
(a) An approximator was placed either on the artery at 1.5 cm from each other under the surgical microscope (x16 magnification). (b) Arteriotomy was made. (c) In the experimental group, the pectin-based hydrogel (box) was injected intraluminally and into the anastomosis site with a 26-gauge injector. Stent effect of the hydrogel preventing the collapse of the lumina of the vessel ends can be observed (arrows). (d) SEM images of the hydrogel) and 3-dimensional illustration of the pectin-lidocaine structures. The red arrows indicate the crystal structure of lidocaine within the hydrogel. (e) Smooth passage of the suture needle through the vessel wall in the gel medium. (f) Graphs showing the effect of the hydrogel on the anastomosis time in arteries and veins. (g) Graphs illustrating the evaluation of vessel diameters in the hydrogel and control groups before and after anastomosis in arteries and veins. (h) Demonstration of the vessel diameter increase in the femoral artery five, ten, and 15 min following hydrogel application *p < 0.05, **p < 0.01, ***p < 0.001, ns indicates no significant difference.

The mean duration of femoral artery anastomosis (21.77 ± 4.79 min) in the control group was found to be longer than the gel group (20.6 ± 4.26 min), but it was not statistically significant (p = 0.65) (Video S3). In terms of femoral vein anastomosis times, the mean anastomosis time in the gel group (22.32 ± 6.03 min) was significantly shorter than the control group (29.07 ± 10.27 min) (p = 0.002). No statistically significant difference was found in perioperative or first-week patency rates between arteries and veins of the gel and control groups (Figure 4f and Table S3). In a study on rat aorta anastomosis using tissue adhesive, the duration of arterial anastomosis was reported as 8.06 min in the experimental group using poloxamer gel, compared to 47.3 min in the control group using conventional sutures.^7^ Similar results were obtained in other studies that focused on the effect of poloxamer hydrogels in arterial anastomoses.^22,23^ The duration of arterial anastomosis performed with traditional sutures varied in different studies, ranging from 47 to 15 min. These discrepancies may be attributed to the choice of artery and its caliber, different time points defining the anastomosis duration, and surgical experience. Performing microvascular anastomosis procedures in veins is technically much more demanding, as they are structurally thinner than arteries, lack the media muscle layer, are fragile, and have a tendency for luminal collapse (Figure 4a-d). The average time for femoral vein anastomosis time was reported to be 38.4 min in a study conducted by Pruthi et al., who emphasized that this period may be even longer in the hands of inexperienced surgeons.^24^ For this reason, we find it very valuable that it has been shown in our study that vein anastomosis can be performed significantly faster and easier in a gel medium. The fact that the pectin hydrogel we designed has a viscosity that can easily enter the lumen of the vein, that the ends of the veins are completely in the gel, thus preventing the collapse of the lumens and that it is almost suspended in the air in the gel has facilitated and accelerated the anastomosis (Figure 4e).

#### 3.5.2 Assessment of Lidocaine Release

We first determined the effectiveness of lidocaine release in arteries and veins in gel medium by measuring the diameter changes before and after anastomosis. Arterial diameters significantly increased in both the gel and control groups compared to before and after anastomosis (p = 0.005). However, the diameter changes before and after arterial anastomosis were significantly higher in the gel medium compared to the control group (*p* < 0.001). For veins, the diameter changes before and after anastomosis significantly increased in the gel group (p = 0.001). The diameter of the vein was significantly enlarged in the gel medium, and although the change in the diameter of the vein in the control group was still significant, it was not as much as the one in the gel group (p = 0.027). Moreover, the enlargement of the vein diameter in the gel medium was found to be significantly higher than the enlargement in the control group (p = 0.001) (Figure 4g, Table S4). This effect was also observed and recorded in veins, which was not reported previously in the literature.

It was observed that the diameter of the femoral artery significantly increased with vasodilation at the fifth, tenth, and 15th minutes after the gel application (p = 0.005, *p* < 0.001, *p* < 0.001, respectively). We observed that the first effect appeared at the fifth minute and the arterial diameter increased in the gel medium for 15 min (Figure 4h, Table S5). Yokoyama et al. evaluated the effectiveness of single and continuous lidocaine administration in a rat model. They showed that the increase in arterial flow velocity started at the second minute, reached a maximum at the ninth minute, and continued to increase until the 15th minute when the measurements were terminated. Thus, we demonstrated that lidocaine at a dose similar to the 4% form designed to be released in the gel can effectively vasodilate femoral arteries and veins similar to the findings observed in studies of Yokoyama et al.^65^

#### 3.5.3 Histological and Immunohistochemical Analysis

**Figure 5:**
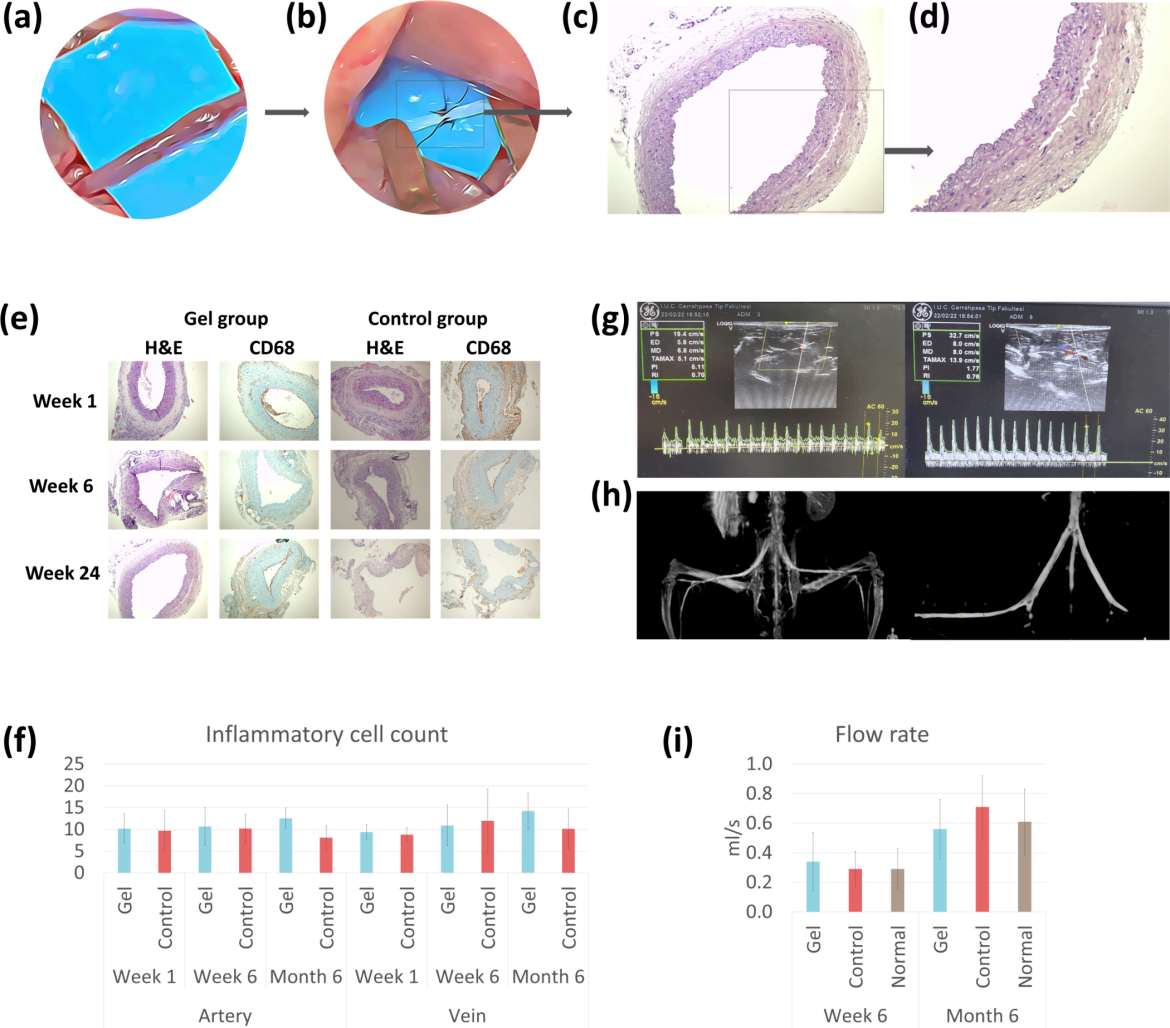
Illustration demonstrating an intact vessel (a) and following anastomosis (b). Black suture threads are visible, which are considered foreign bodies. At the end of the sixth month, a normal arterial cell wall can be observed under ×200 (c) and ×400 (d) magnification. (e) Images of macrophages, giant cells, and inflammatory cells in ×200 microscope magnification with CD68 and H&E staining were observed in the femoral artery at 1 week, 6 weeks, and 6 months. (f) Graphical demonstration of the inflammatory cell counts in arterial (left) and venous (right) vessel walls at different time points (g) Doppler USG measurements of femoral artery flow rate at 6 weeks (left) and 6 months (right) in the gel group. Also, patency and diameter measurements of the femoral artery and vein. (h) Angiographical images of the same subject demonstrating consistent results in patency. (i). Bar charts comparing the flow rates in the gel and control groups with respect to the normal values obtained from animals where no interventions were performed *p < 0.05, **p < 0.01, ***p < 0.001, ns indicates no significant difference.

In microsurgery, a foreign body reaction is expected to occur against microstitches, which are nonabsorbable permanent foreign bodies in the vessel wall used during vascular anastomosis (Figures 5a and b).^7,16,23^ The mean numbers of peri-vascular giant cells in the femoral artery and vein groups were similar to the perivascular response without gel in anastomoses performed in gel medium at week 1, week 6, and month 6 (Figures 5e and f and Figure S18). Similarly, Smith et al. reported that perivascular inflammation in the first week of arterial anastomosis in a drug-free and peptide-based hydrogel medium with a very similar method to our study was not different from the control group and only the foreign body reaction to the sutures was similar in both groups.^16^ Likewise, when total inflammatory cell counts were analyzed; very similar results were observed in the perivascular area for arteries and veins in anastomoses with and without gel at first and sixth week. At the sixth month , inflammatory cells increased in the gel group compared to week 6, whereas they decreased in the control group, but there was still no statistically significant difference (Table S6, Figure 5f). In their study, Chang et al. demonstrated that the number of giant cells, inflammatory cells, and CD68 macrophages increased in the sutured anastomoses compared to the first week, and they were still present but gradually decreased in the sixth month and first year.^7^ In our study, we found that the mean number of giant cells around the vessel wall was very similar in the first week, sixth week, and sixth month, regardless of the use of the gel. As far as we know, there is no study in the literature evaluating perivascular inflammation for microvascular anastomosis for veins. Additional histopathological examination parameters that were compared in our study, such as EVG and Reticulin stains for vessel wall fibrosis and vessel wall integrity in the late sixth month, showed that vascular wall disruption and an increase in reticulin fibers due to microsutures were found in both arteries and veins in the gel and nongel groups, but there was no significant difference between them (Table S7, Figure S19, and S20). In addition, both groups showed similar immunohistochemical staining against CD31 antibodies, indicating statistically insignificant endothelial integrity between the gel and control groups (Figure S21 and S22). Chang et al. also stated that there may still be irregularity due to sutures in the arterial wall in classical sutured anastomoses with EVG at the end of the first year.^7^ As a result, we observed for the first time in our study that perivascular inflammation in arteries and veins, fibrosis with reticulin fiber increase in the vessel wall, vessel wall integrity, and endothelialization showed similar behaviors in gel and gel-free medium and there was no difference, foreign body reaction to nonabsorbable suture material and inflammatory response caused by surgical trauma and related fibrosis formation did not increase significantly with pectin hydrogel (Figure 5c and d).

#### 3.5.4 Vascular Imaging with Doppler Ultrasound and CT Angiography

After microvascular anastomosis, measuring arterial patency and flow velocity is crucial. Methods used include Doppler US (Figure 5g), CT angiography, MRI angiography, conventional angiography (Figure 5h), and optical coherence tomography.^7,16,23,86^ Measuring small vessels such as the femoral artery in rats and mice is technically difficult, particularly with Doppler US and arterial catheterization.^62,63,86,87^ In the literature, femoral artery flow velocities, patency, and artery diameters were examined in a few studies with radiologic imaging methods after microanastomosis in rats and generally evaluated in the early first week and the late sixth week.^17,27^

In our study, arterial patency was found to be 100% in both the control and gel groups at the sixth week and sixth month, while there was a vein thrombus at the sixth week and sixth month in the gel for vein groups. However, there was no significant difference in the sixth week and sixth month vein patency rates in the gel groups when compared with the control groups (Table S8).

The mean femoral artery diameter measured by Doppler USG at week six was 1.18 ± 0.3 mm in the gel group, 1.19 ± 0.32 mm in the control group, and 1.16 ± 0.25 mm in the left-sided normal (without any surgical intervention) femoral artery group. There was no statistical difference between these values (p = 0.973) (Table S9). Similarly, the mean femoral vein diameters were not significantly different between the gel (1.16 ± 0.32 mm), control (1.17 ± 0.45 mm), and normal (1.21 ± 0.34 mm) groups (p = 0.962) (Table S9). Although the mean femoral artery flow velocity in the gel group (0.34 + 0.2 mL.s-1) was slightly higher compared to the control group (0.29 + 0.12 mL/s) and normal group (0.29 + 0.14 mL/s), there was no statistically significant difference (p = 0.785) (Figure 5i, Table S9). In the sixth month, the mean femoral artery diameter of the gel (1.44 ± 0.18 mm), normal (1.43 ± 0.21 mm), and control (1.44 ± 0.14 mm) groups were almost identical (p = 0.98) (Table S9). The mean femoral vein diameters were also not significantly different between groups, with values of 1.37 ± 0.22, 1.47 ± 0.21 and 1.36 ± 0.22 mm, respectively, compared to the normal group (p = 0.54)) (Table S9) Although the mean femoral artery flow velocity was higher in the control group (0.71 ± 0.21 mL/s), it was not statistically significantly different from the gel (0.56 ± 0.2 mL/s) and normal (0.61 ± 0.22 mL/s) groups (p = 0.421) (Figure 5i, Table S9). Both Chang et al. and Ozer et al. found no difference in artery patency and flow rates when comparing the special polymer microanastomosis method without sutures to the sutured method in rat aortic arteries and the use of heparin-loaded gel, respectively.^7,23^ While late femoral vein diameter measurements after microvascular vein anastomosis have rarely been examined in the literature, vein patency assessment has been evaluated only by direct observation in very few studies and objective examination with radiologic methods has never been performed.^24,88,89^

## 4 Conclusions

This study aimed to develop an injectable pectin hydrogel with the ability to release the vasodilator lidocaine and to prevent collapse of vessel lumens, and hence, the ability to facilitate microsurgical suturing. With this aim, three complementary approaches were employed, i.e. computational calculations, hydrogel development, and *in vivo* application sharing mutual feedback throughout the study. In the computational part involving molecular dynamics and quantum mechanics calculations, the suitability of using lidocaine in the pectin hydrogel was elucidated by focusing on molecular interactions dominating the drug release behavior of the hydrogel. Then, guided by the computational findings, a pectin-based hydrogel was designed with lidocaine release properties that was injectable, stable during surgery, self-healing, and self-degradable after surgery. In addition, our preliminary studies have shown that this hydrogel does not cause significant changes in late-stage imaging and histopathological examinations. To the best of our knowledge, we developed an injectable pectin hydrogel, with numerous desirable properties, that can be used as a facilitator in microlevel arterial, lymphatic, and especially venous anastomoses. Further studies are needed to establish the efficacy of this hydrogel for clinical use.

## Data Availability

Data will be made available on request.

## Supporting Information Available

Molecular dynamics and quantum mechanics calculations, structural and morphological characterization of the hydrogel, lidocaine release behavior of the hydrogels, rheological analysis and *in vivo* test results (PDF)
Video S1. Assessment of lidocaine-releasing properties of the gel on the left sided femoral artery under surgically induced vasospasm (MP4)
Video S2. Arterial image obtained from CT angiography (MP4)
Video S3. Microsurgical process of femoral artery anastomosis in the gel medium (MP4)
