# Supplementary material for: Innovative Use of an Injectable, Self-Healing Drug-Loaded Pectin-Based Hydrogel for Micro- and Supermicro-Vascular Anastomoses
Source: Biomacromolecules. 2024 Jun 27;25(7):3959–75. doi: 10.1021/acs.biomac.4c00102 (PMC11238333; doi:10.1021/acs.biomac.4c00102)
Supplement: Supplementary file 1 — bm4c00102_si_001.pdf [file bm4c00102_si_001.pdf]

Supporting Information

Innovative use of an injectable, self-healing drug-loaded pectin-based hydrogel for micro- and supermicro vascular anastomoses

Banu Kocaaga[1], Tugce Inan[1], Nesrin İsil Yasar[2], Can Ege Yalcin[3], Fethiye Aylin Sungur[2], Ozge Kurkcuoglu[1], Anil Demiroz[3], Hasan Komurcu[4], Osman Kizilkilic[5], Servet Yekta Aydin[3], Ovgu Aydin Ulgen[6], Fatma Seniha Güner[1,7,], Hakan Arslan[3,*]*

[1] Istanbul Technical University, Department of Chemical Engineering, Maslak 34469 Istanbul, Turkey

[2] Istanbul Technical University, Informatics Institute, Computational Science and Engineering Division Maslak 34469 Istanbul, Turkey

[3] Istanbul University-Cerrahpasa, Cerrahpasa Medical Faculty, Department of Plastic, Reconstructive and Aesthetic Surgery, Istanbul 34089 Turkey

[4] Balat Or-Ahayim Hastanesi, Department of Plastic, Reconstructive and Aesthetic Surgery, Istanbul 34087 Turkey

[5] Istanbul University-Cerrahpasa, Cerrahpasa Medical Faculty, Department of Interventional Radiology, Istanbul 34098 Turkey

[6] Istanbul University-Cerrahpasa, Cerrahpasa Medical Faculty, Department of Pathology, Istanbul 34098 Turkey

[7] Sabancı University Nanotechnology Research and Application Center, Istanbul 34956 Turkey

1. Molecular dynamics and quantum mechanics calculations

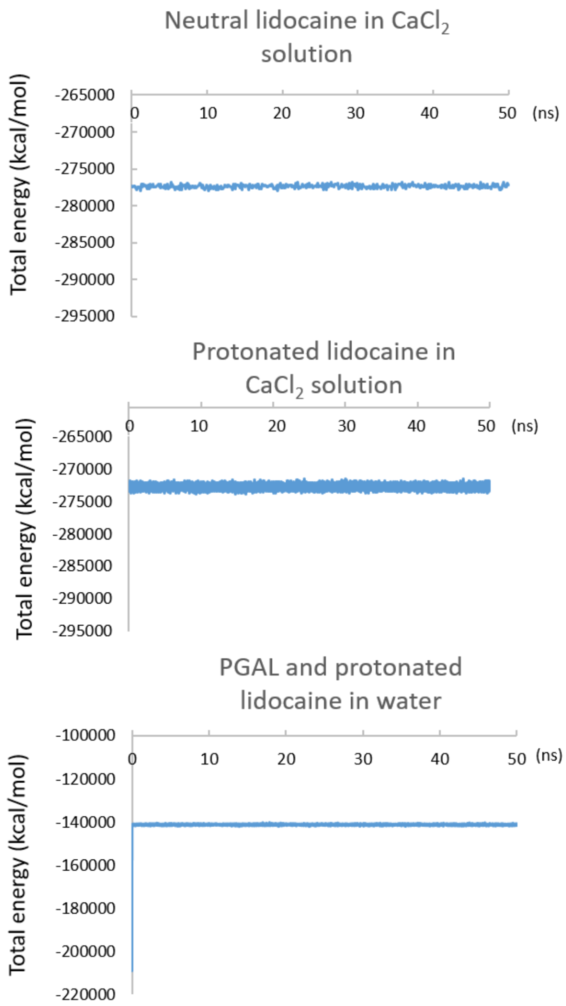

Figure S1. Total energy profiles of the systems over the production runs of the molecular dynamics simulations.

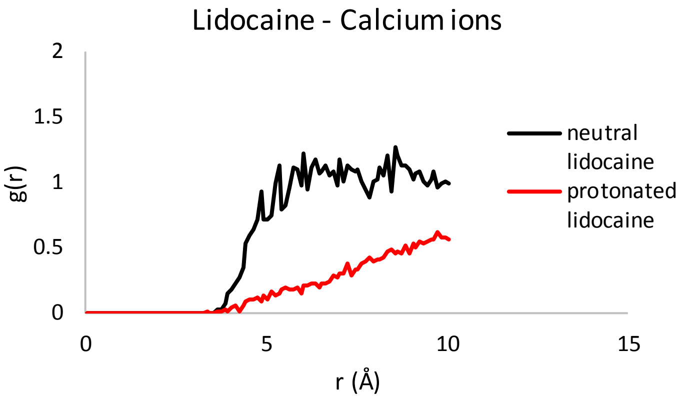

Figure S2. Radial distribution function g(r) analysis between Ca^{2+} ions and lidocaine molecules for the model systems neutral lidocaine in $CaCl_2$ solution and protonated lidocaine in $CaCl_2$ solution.

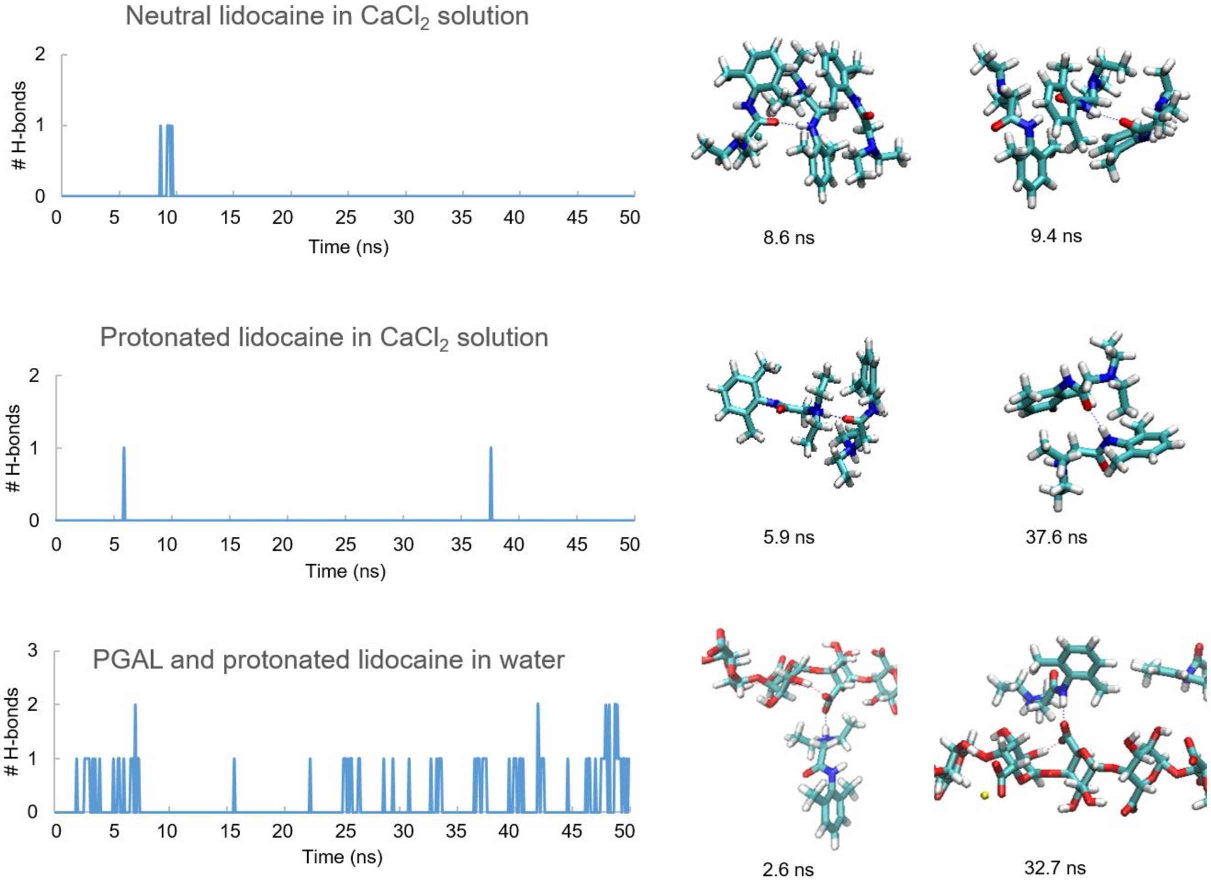

Figure S3. The hydrogen bond analysis of the systems investigated with molecular dynamics simulations. Snapshots at the right panel show the hydrogen bond interactions (in dashed blue line). Hydrogen bond interactions were analyzed using the Hydrogen Bonds analysis tool of VMD[1]. The hydrogen bond was defined for donor-acceptor distance of 3.0 Å and an angle of 20°.

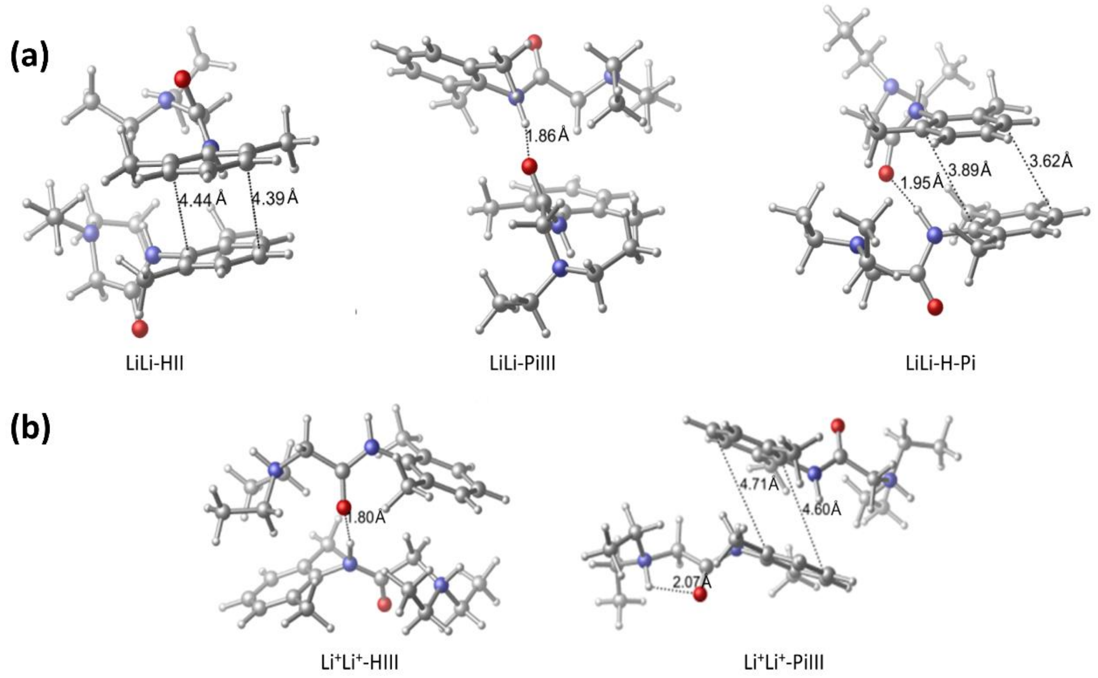

Figure S4. Best interaction energy complexes obtained for (a) neutral lidocaine-lidocaine interactions, (b) protonated lidocaine-lidocaine interactions.

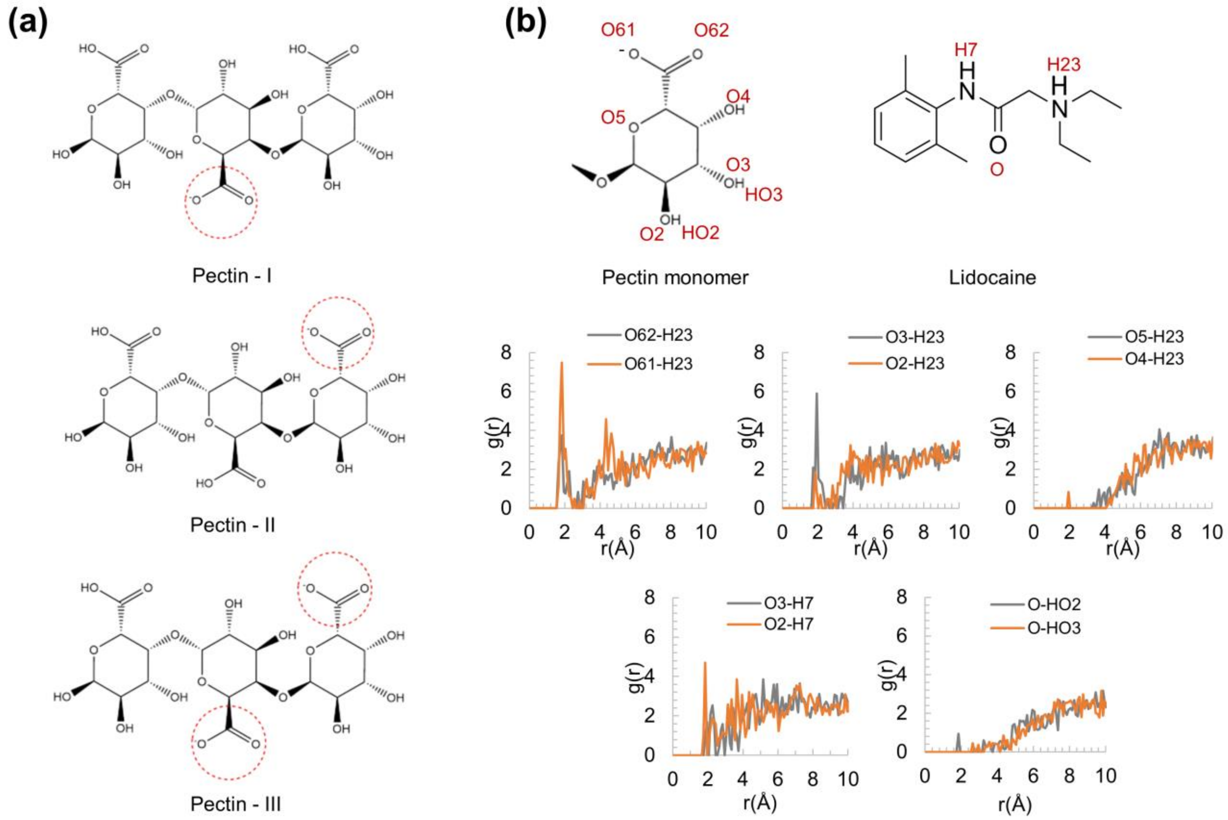

Figure S5. (a) Pectin structures with three different protonation states. Deprotonated carboxyls are shown with a red circle. (b) Radial distribution function g(r) for lidocaine-pectin interactions during Molecular Dynamics simulations. Atom-pairs for g(r) plots are indicated by red labels.

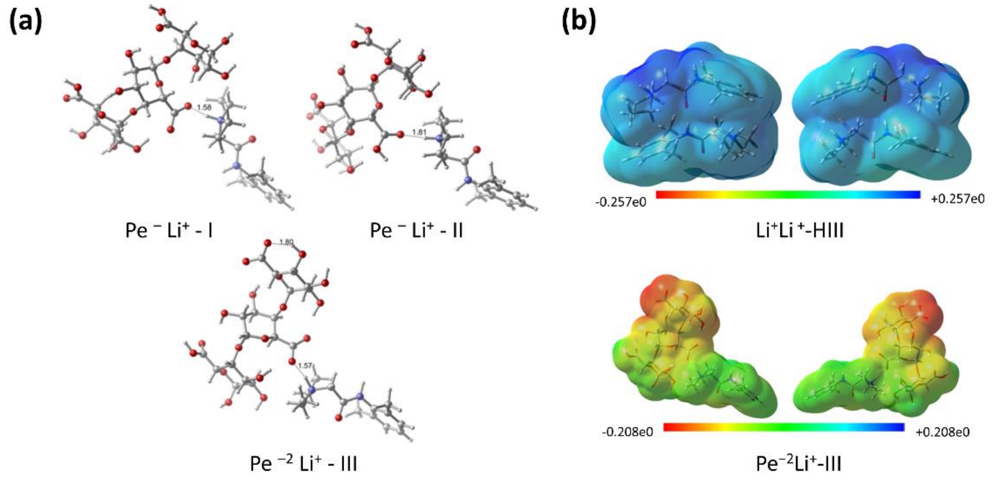

Figure S6. (a) 3D representation of structures showing interaction between Pectin-Protonated Lidocaine. (b) ESP charge distribution map of the structures Li+Li +-HIII and Pe−2Li+-III

2. Structural and morphological characterization of the hydrogel

The Fourier transform infrared (FT-IR) spectrum analysis was used to investigate the physicochemical interactions between pectin, CaCl2, and lidocaine in hydrogel films. FT-IR spectra of pure pectin, 4P-2 hydrogel and lidocaine are shown in Figure S7. The peaks in the 2920-2990 cm^{-1} region represent C-H aliphatic stretching bands. The characteristic peak of pure pectin which is seen at 1677 cm^{-1} (asymmetric vibration of carboxylate ions) in the spectra shifted to 1672 cm^{-1} for 4P-2 hydrogel [2,3]. The characteristic peak of the lidocaine molecule, which belongs to -C-CH3 methyl groups, is observed at 1474 cm^{-1} in the drug-loaded pectin hydrogel (4P-2). The

peak at 1271 cm^{-1}, which arises from the tertiary amine structure of lidocaine, is also observed at 1274 cm^{-1} in the pectin hydrogel structure [4].

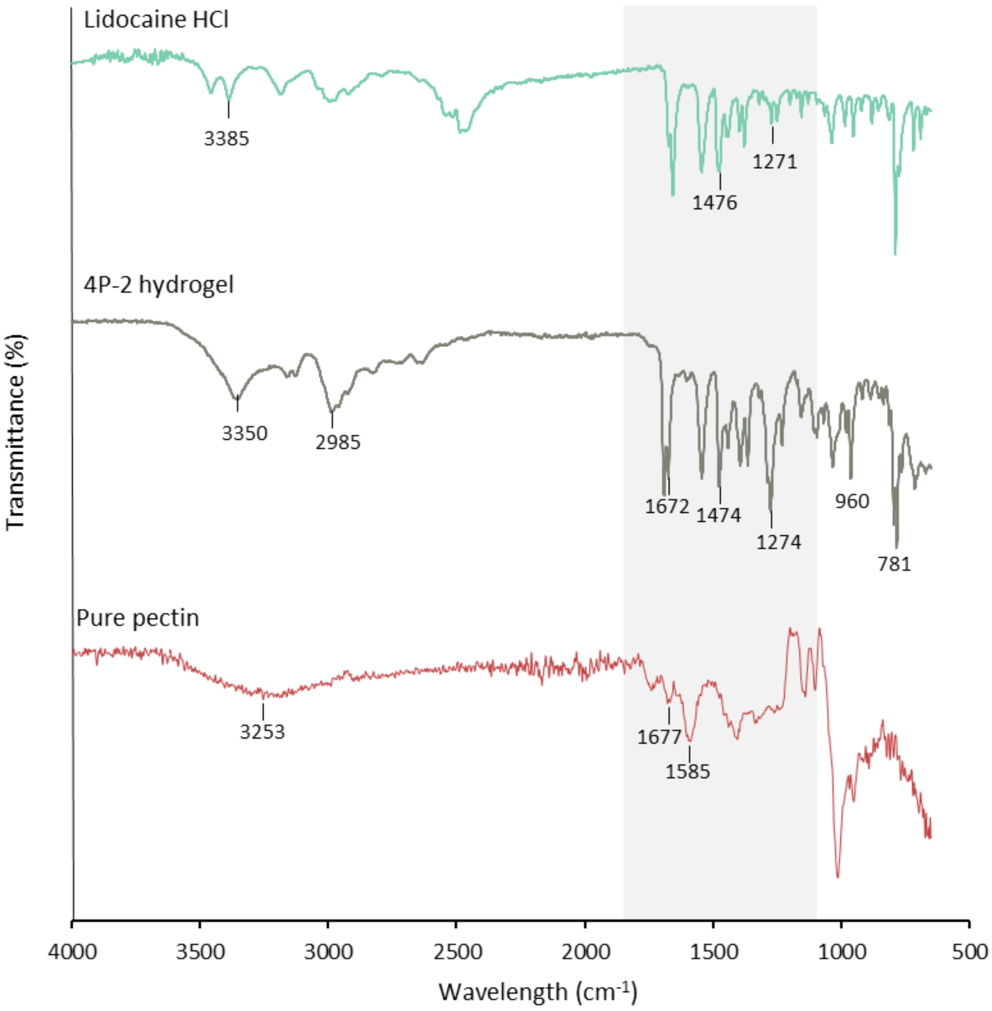

Figure S7. FTIR spectra of pectin, 4P-2 hydrogel and lidocaine

The SEM images of the dry 4P-2 hydrogel are shown in Figure S8. The intricate pore architecture within the hydrogel is clearly visible in Figures S8a-c. During 4P-2 hydrogel synthesis, the drug-loaded pectin solution (DLP) containing 4% pectin and the crosslinking solution (CLS) made with 1% pectin and a dilute $CaCl_2$ solution were mixed with each other. The resultant hydrogel

structure, characterized by a rugged surface with overlaid layers, is likely a consequence of the differential concentrations in the two mixed solutions. In this process, the $CaCl_2$ crosslinker phase with its dilute pectin solution may have filled the pores within the lidocaine-loaded phase, thereby altering the expected porous structure. Additionally, the drying method plays a crucial role; the hydrogel was air-dried rather than freeze-dried, a process which typically leads to larger pore sizes due to the slower removal of water, further deviating from the conventional porous hydrogel structure. Moreover, the SEM images of the powdered lidocaine in Figures S8e-h are in accordance with the literature [5,6]. Specifically, lidocaine has been shown to form visible crystalline structures in various formulations. For example, studies on lidocaine tablets and on formulations combining lidocaine with hydroxypropyl-β-cyclodextrin (HP-β-CD) and sodium saccharin have successfully demonstrated the crystalline structure of lidocaine using SEM imaging. In these cases, the crystalline structures are typically larger due to the aggregation of lidocaine molecules, often influenced by the presence of other components within the sample which facilitate the formation of such aggregates [6].

As highlighted by the red arrows, the crystal structures of lidocaine present in the hydrogel can also be observed in Figures S8c, d. Although lidocaine is a relatively small molecule with a molecular weight of 234 g/mol, the structures identified in the SEM images appear larger. This discrepancy might be attributed to the inter- and intra-molecular interactions of lidocaine molecules, which were elucidated in our computational studies (Figure S4). These interactions may result in the formation of lidocaine clusters within the hydrogel matrix. Consequently, the SEM images may effectively demonstrate these lidocaine clusters, providing visual evidence of their presence and confirming their significance in the structure of the hydrogel.

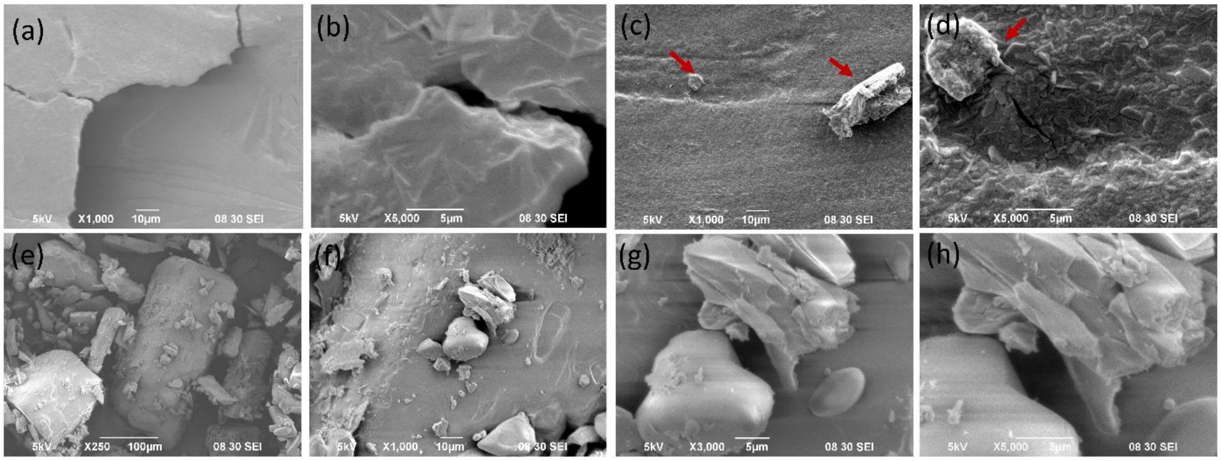

Figure S8. SEM images (a-d) 4P-2 hydrogel, (e-h) Lidocaine HCL. The red arrows in (c) and (d) indicate the crystal structure of lidocaine within the hydrogel.

3. Lidocaine release behavior of the hydrogels

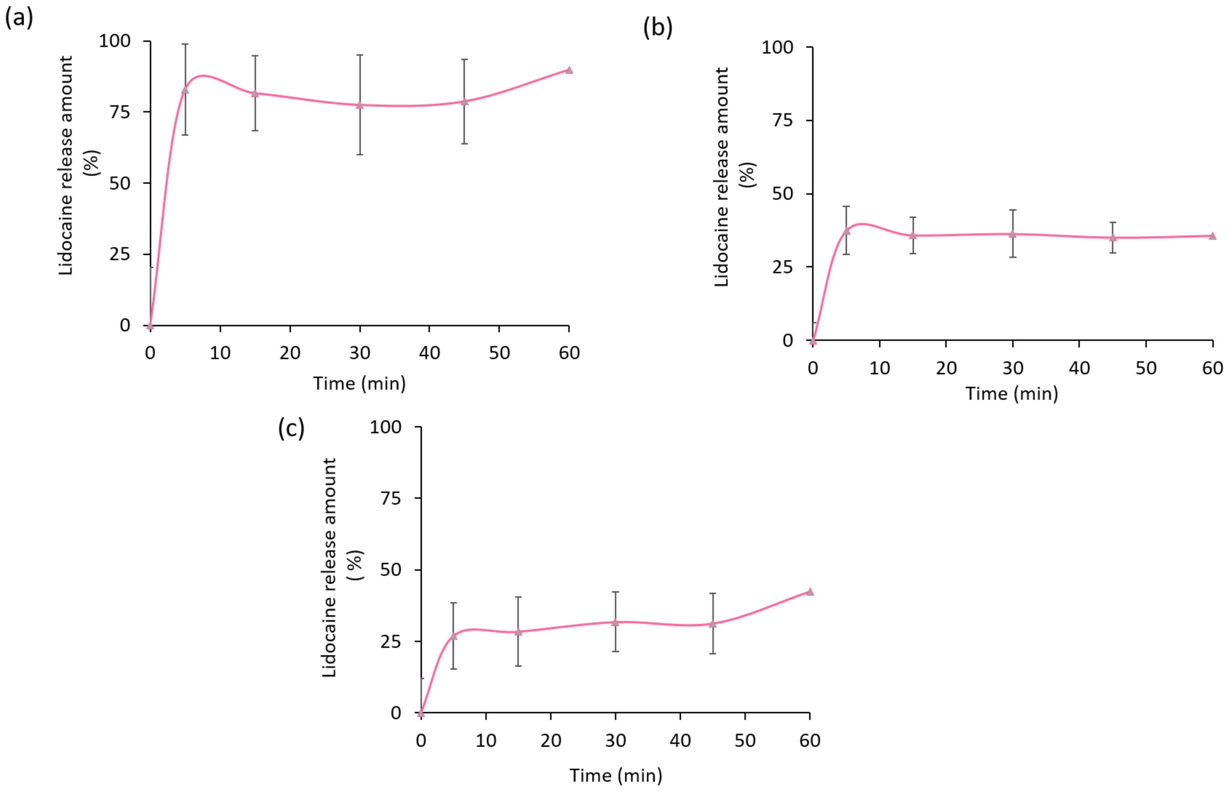

Figure S9. Lidocaine release kinetic of 4P-2 hydrogel in various environments: (a) Isotonic (pH 5.5), (b) Citrate buffer (pH 5.0), (c) PBS (pH 7.4).

4. Rheological analysis

Time sweep analysis: The initial formation of the 4P-2 hydrogel (gelation point) which occurs when it transitions from a liquid-like to a solid-like behavior was detected by using time sweep analysis. The storage (G') and loss (G") moduli at the gelation point were equal. As shown in Figure S10, the resulting G' and G" data were plotted as a function of time.

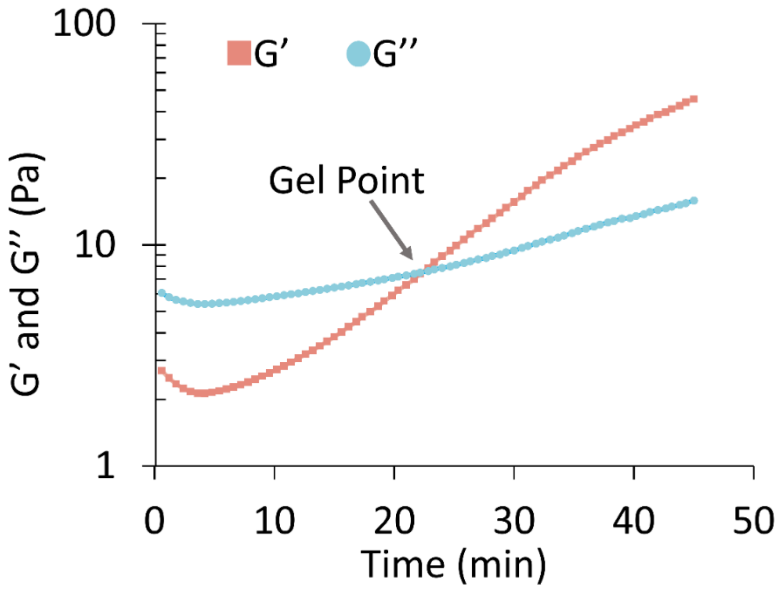

Figure S10. Time sweep analysis of 4P-2 hydrogel.

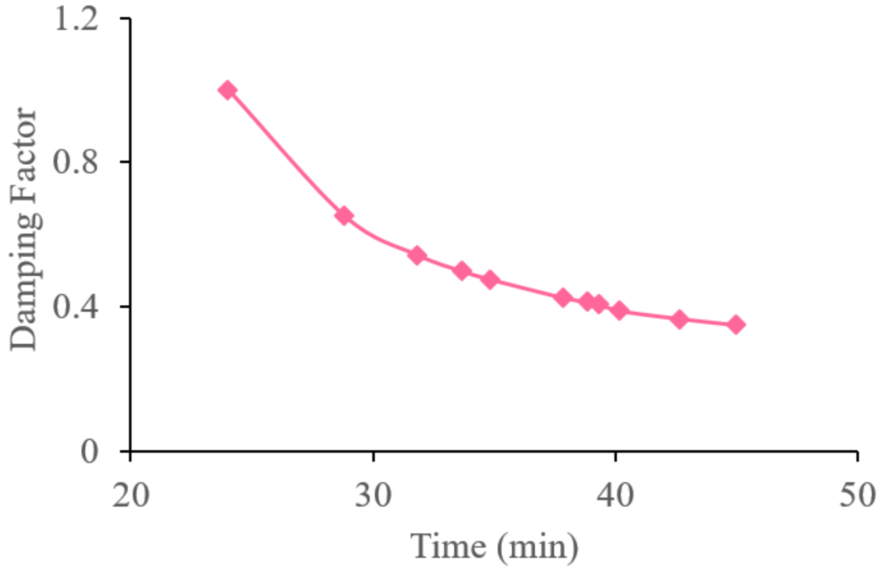

Figure S11. Damping factor of 4P-2 as a function of time.

Viscosity of the hydrogel:

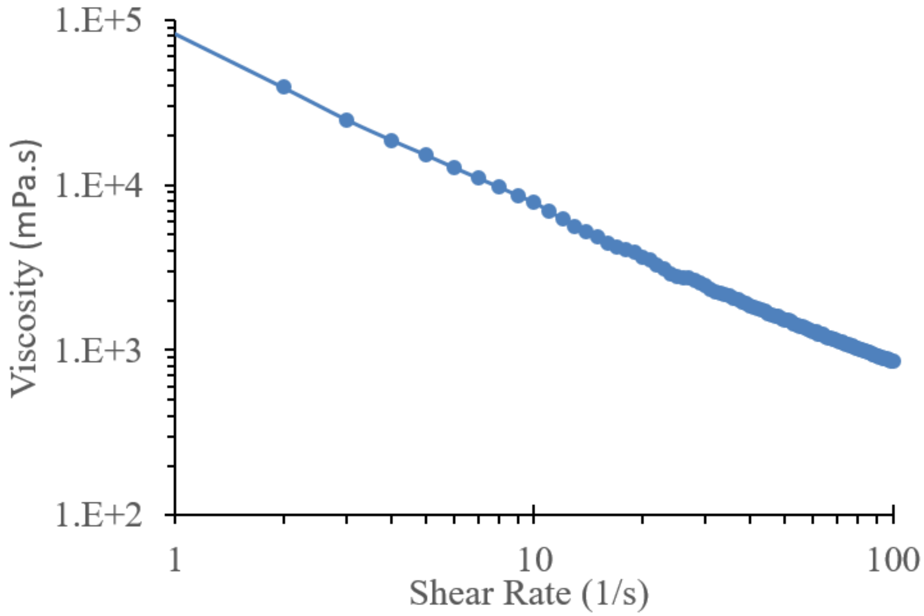

Figure S12. Viscosity of 4P-2 hydrogel

The data illustrated in Figure S12 reveal that as the shear rate increases, the viscosity decreases, demonstrating shear-thinning behavior as described by the Oswald-de Waele (power law) model. This behavior is supported by the flow behavior index, n, is less than 1[7], at a shear rate of 1 s^{-1}, the viscosity is 82925 mPa·s. This value significantly decreases to 859.86 mPa·s when the shear rate is increased to 100 s^{-1}, highlighting the material's dynamic response to varying shear rates. The considerable reduction in viscosity under higher shear rates can be attributed to the disruption of hydrogen bonds and van der Waals interactions, leading to the desired shear-thinning behavior. This property is particularly advantageous for injectable materials, ensuring ease of administration while maintaining structural integrity[8].

Amplitude sweeps analyses: For injectable hydrogels, a strain sweep analysis can help determine the gel's elastic modulus, yield stress, and strain which are crucial for assessing the gel's ability to withstand mechanical stresses and recover its original shape after deformation [9–11]. For this reason, amplitude sweep analyses were conducted to determine the linear viscoelastic region and apparent yield stress or strain at the point where G' equals G'' (G'=G''), a threshold necessary to initiate flow, as illustrated in Figure S13 [10,12,13]. The results in Figures S13a and b demonstrate how the viscosity of the hydrogel varies as a function of shear stress and strain, respectively. The data show that once the hydrogel is subjected to a critical point of shear stress or strain at the flow point (σ_f,γ_f), its viscosity decreased significantly. Additionally, Figures S13c and d present the variations of G' and G'' with respect to shear stress and strain. Initially, at relatively low stress or strain levels, the amplitudes exert minimal influence on G' and G'', with G' predominating over G'' across all hydrogels, signifying a highly stable structure. However, surpassing a critical strain or shear stress threshold leads to an intersection of the G' and G'' curves, where G' falls below G'',

denoting the hydrogel's structural collapse and its transition from a gel state to a sol state (Figures S13c and d).

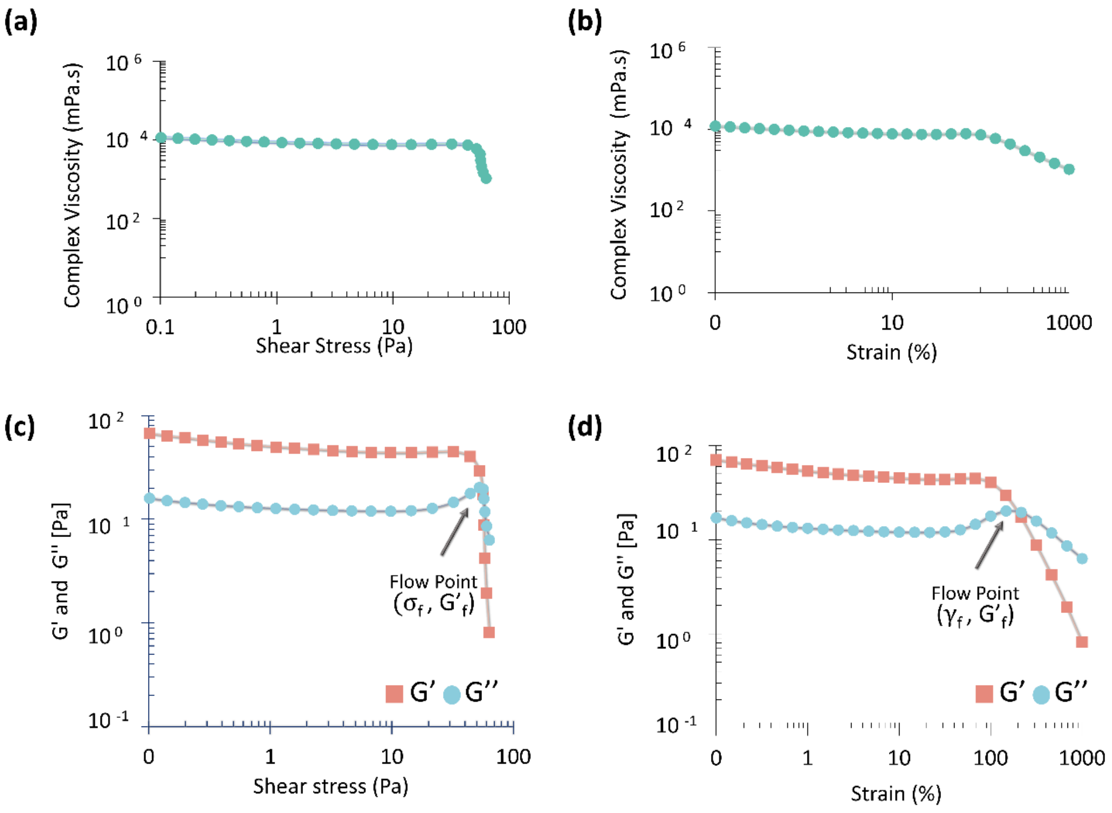

Figure S13. Amplitude sweep analyses of 4P-2 hydrogel

Degradation of the hydrogel at the end of the anastomosis operation: We assesed the spontaneous gel network's decomposition (gel-sol transition) after suturing. For this purpose we conducted a frequency sweep analysis for both 4P-2 and 4P-2_d hydrogels (Figure S14).

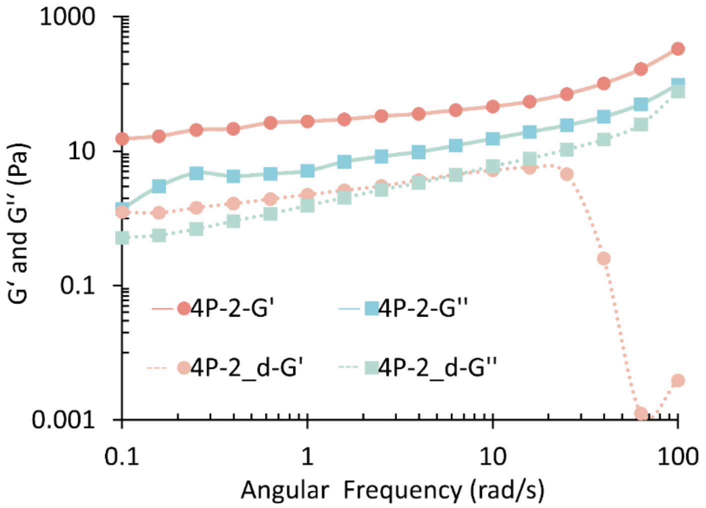

Figure S14. Frequency sweep analyses of both 4P-2 and 4P-2_d hydrogels. Here 4P-2_d hydrogel is presented with dash lines.

Creep and Recovery Analysis: The viscoelastic behavior of 4P-2 was analyzed by conducting creep-recovery test. In this test, the gel gradually deforms under applied stress, exhibiting both elastic (recoverable) and viscous (non-recoverable) characteristics. The sample responds to the stress by showing increasing strain until it fails, particularly if the strain is sufficient to disrupt the gel structure. Upon releasing the applied stress, the recovered strain displays an immediate elastic rebound, a fully recovered elastic portion, and a permanently viscous component. The findings from the creep test offer valuable insights into the enduring performance of injectable hydrogels under prolonged loads, mimicking conditions encountered in vivo [14].

The creep test caused a time-dependent increase in strain, comprising elastic and viscous contributions. For illustrative purposes, the creep phase in Figure S15 is divided into three main zones, each exhibiting distinct components: (a) instantaneous elastic deformation, (b) delayed

elastic deformation, and (c) viscous (Newtonian) flow elements of strain [15,16]. The elastic behavior of the sample can be attributed to the stretching of linkages between structural units. Upon the release of shear stress, the instantaneous portion recovers first, followed by the retardation element, and ultimately, the viscous strain[16].

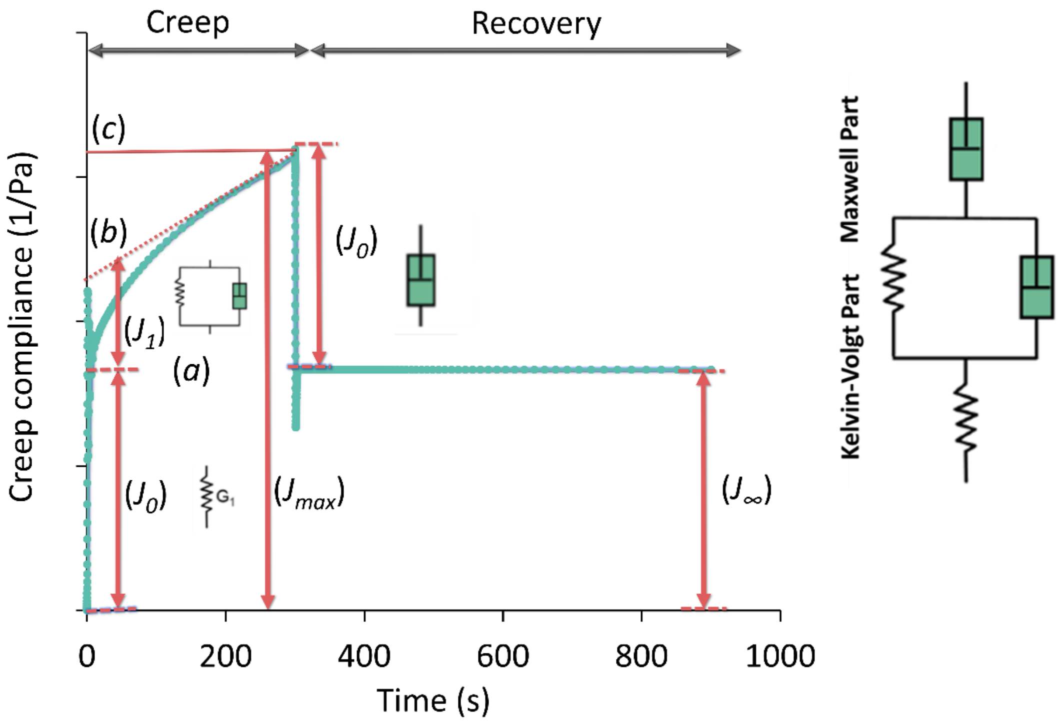

Figure S15. Illustration of a typical creep-recovery curve and dashpot of the Burger model consisted of Maxwell and Kelvin-Voigt models in series.

The response from creep-recovery tests is expressed as creep compliance ($J(t)$), defined as the ratio of measured strain to the applied shear stress according to Equation S1 [16].

$$(t) = (t) / \sigma \qquad \text{(S1)}$$

Here, $\gamma(t)$ represents shear deformation (strain), and σ is the applied constant shear stress. The creep and recovery tests are commonly described through the Burger's model which is a tool for examining the creep-recovery data both inside and outside the lineer viscoelactic region (LVR), comprising a Maxwell element and a Kelvin–Voigt element in series (Figure S15) [17]. The Maxwell element provides instantaneous compliance (spring) and zero shear viscosity to regulate permanent deformation. It indicates the gel rigidity or strength which represents the irreversible damage of strong primary bonds such as ionic and covalent bonds [14]. Kelvin-Voight spring representing the contribution of the delayed elastic (viscoelastic) region to the total creep compliance. It reflects the gel cohesive force or reversible weaker secondary bonds like van der walls [17].

The overall recovery percentage (RC%) of the Burger's model is determined from Jmax and J∞, using the Equation S2 16.

$$RC(\%) = \frac{J_{max} - J_{\infty}}{J_{max}} x \ 100 \qquad \text{(S2)}$$

Here, Jmax represents maximum deformation corresponding to the compliance value once the stress is removed, and $J\infty$ is the compliance for the longest time (Figure S15)[11,18,19]. The recovery, point (c) in Figure S15, is instantaneous and it is made possible by the potential energy of the material.

Figure S16a shows the strain-time and Figure S16b shows creep compliance-time curves. The creep-recovery curve 4P-2 hydrogel exhibited a combination of viscous fluid and solid like viscoelastic component. After the removal of the stress, there was a sharp reduction (from 0.79 Pa-1 to 0.032 Pa^{-1}) in compliance due to a residual and irreversible deformation. 4P-2 hydrogel retianed a residual deformation in agreement with its predominent physical gel character with low cross-linking degree[19]. Calculation using Equation S2 yielded an RC (Recovery Percentage) of 45% (Table S2). This pretty high resistance capacity contributes to elasticity. Notably, this value

exceeds that of the injectable hydrogel commonly used in biomedical applications, which, based on poloxamer, demonstrated a 35% recovery in a recent study focused on creep-recovery experimentation [11].

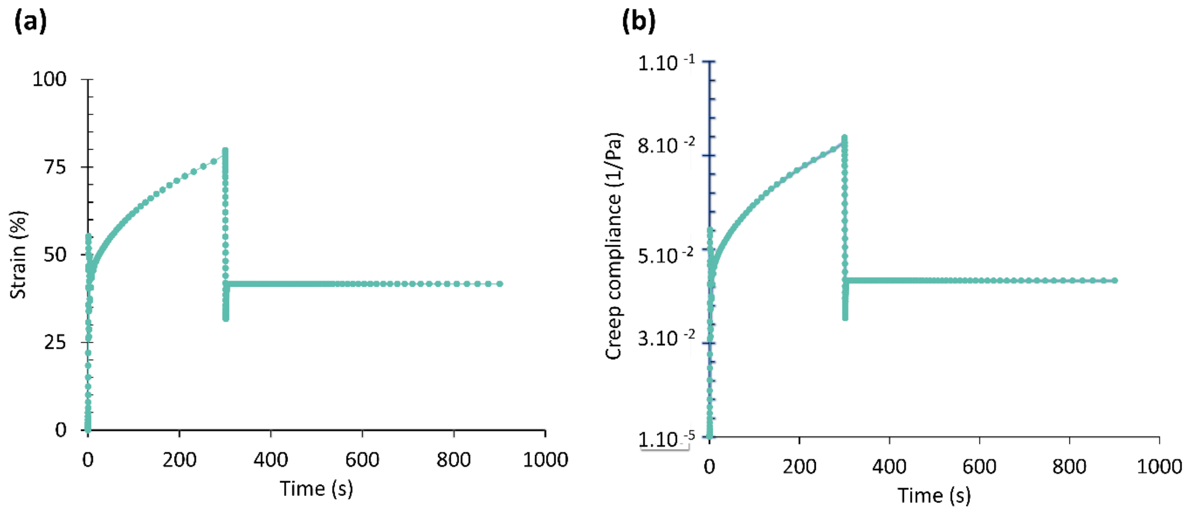

Figure S16. Creep-recovery curves (a) Strain versus time, and (b) Creep compliance versus time.

Tack Test for Adhesion: The tack test involves measuring the force required to separate two surfaces that have come into contact with a material sample. The tack test can be used to quantify these properties by measuring the force gap and force-time values. This analysis is strongly dependent on the bulk rheological properties of the hydrogel as reported in the literature [20]. The results of the tack test analysis are seen in Figure S17. A lower value in either force-gap (measured in energy units; N·mm) (Figure 11a) or force-time (measured in momentum units; N·s) (Figure 11a) during the tack test indicates reduced adhesion[21,22].

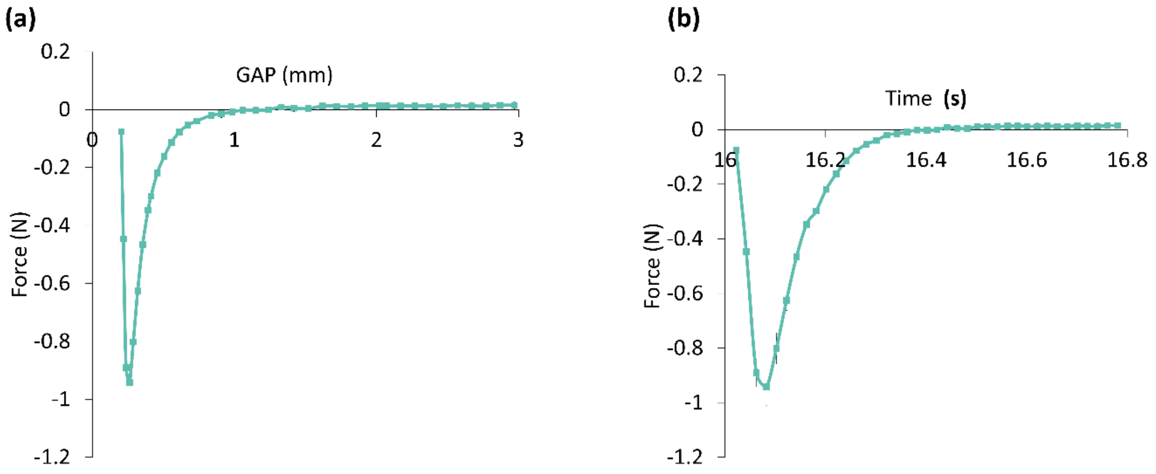

Figure S17. Tack analysis (a) Force-GAP curve, (b) Force-Time curve for 4P-2 hydrogel.

5. *In vivo* test results

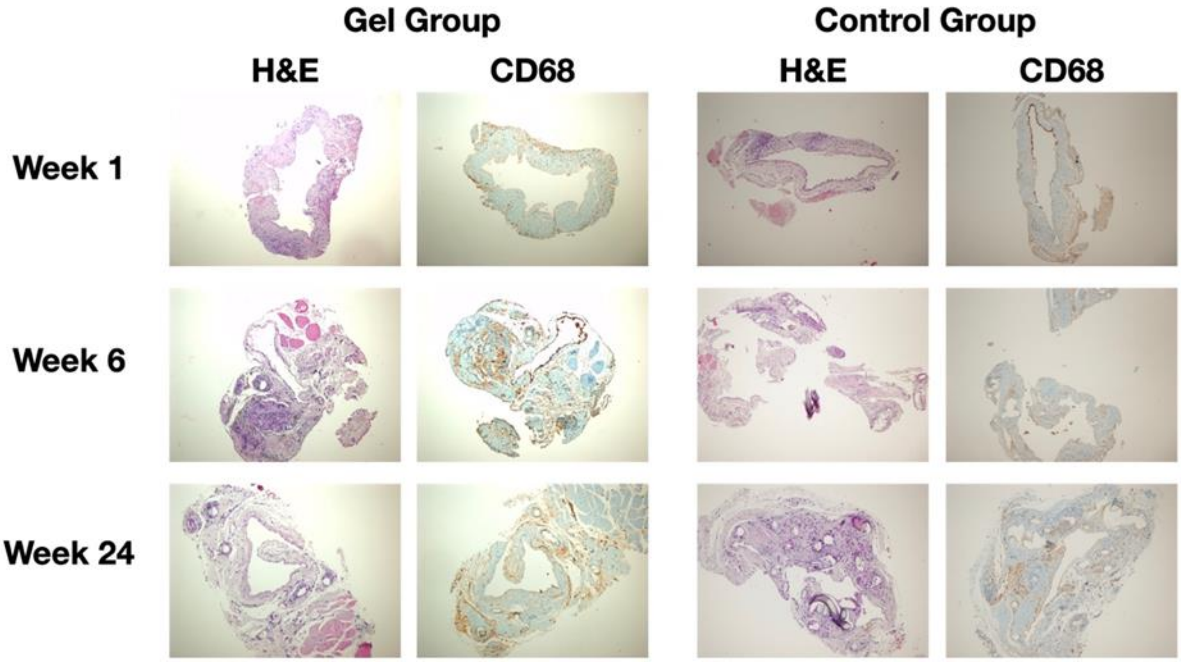

Figure S18. Images of macrophages, giant cells and inflammatory cells in femoral veins at 1 week, 6 weeks and 6 months with CD68 and HE staining at x 100 microscope magnification**.**

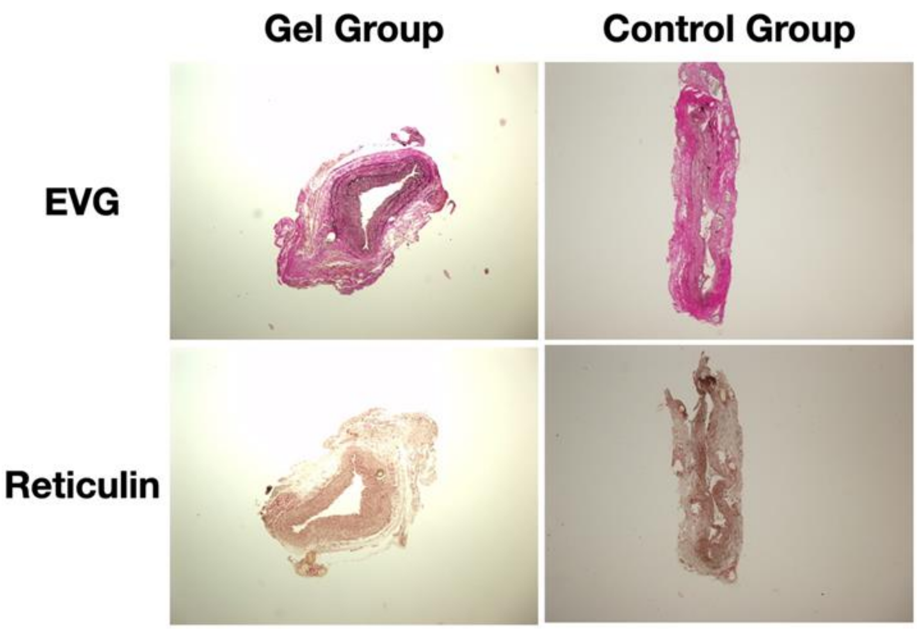

Figure S19. Images of vessel wall fibrosis and integrity in femoral arteries with EVG and Reticulin staining at x 100 magnification.

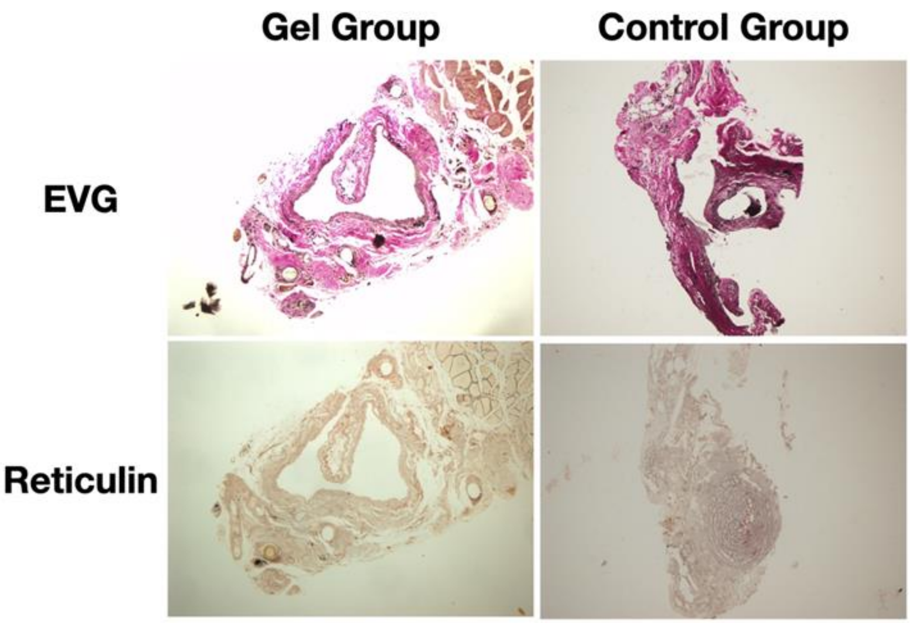

Figure S20. Visualization of vessel wall fibrosis and integrity at x 100 magnification with EVG and Reticulin staining in femoral veins.

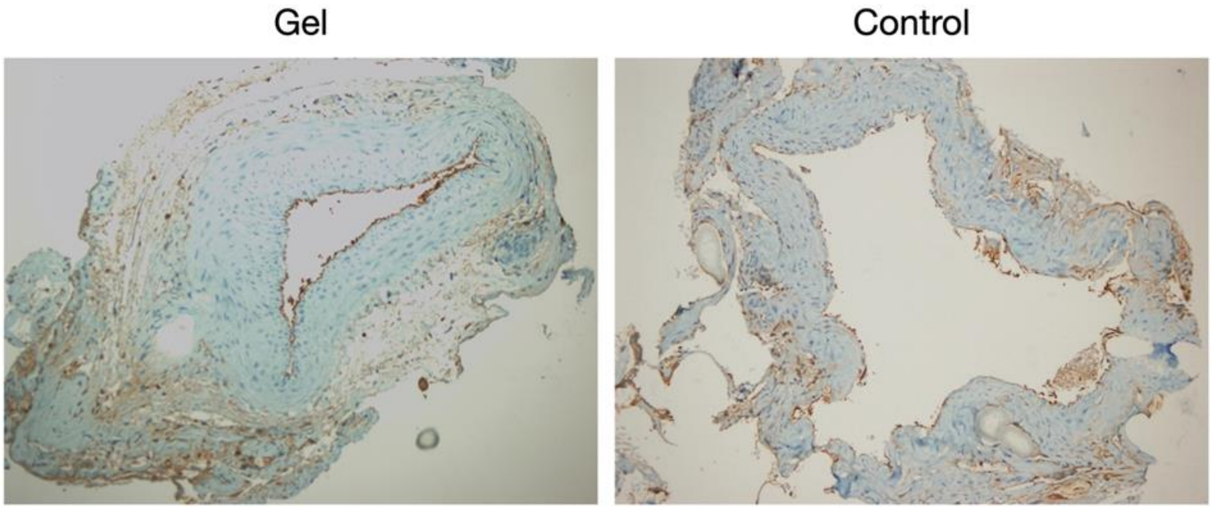

Figure S21. Femoral artery sixth month CD31 endothelial integrity image at x 200 magnification.

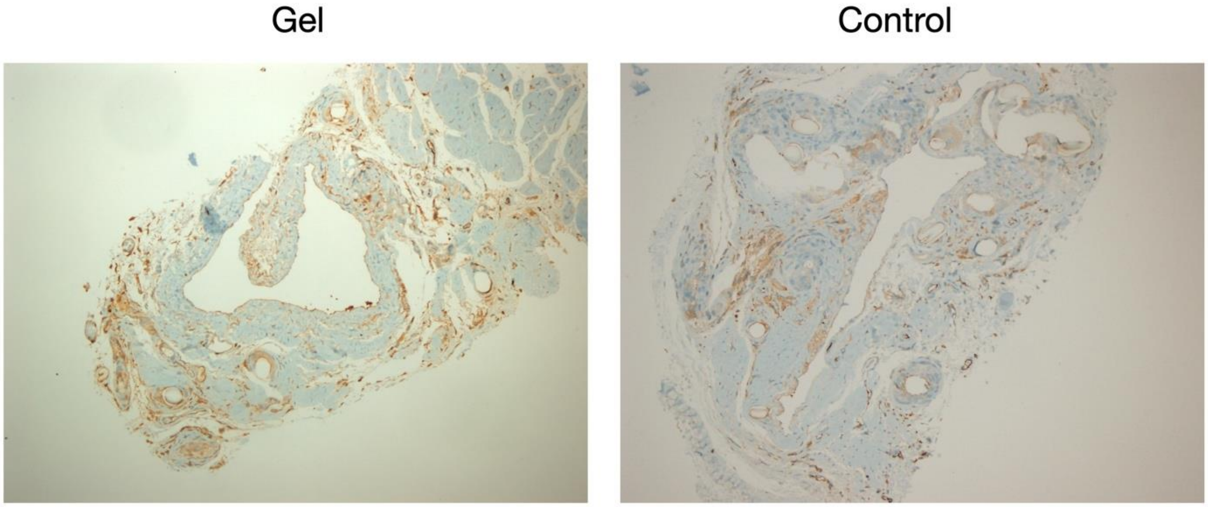

Figure S22. Femoral vein sixth month CD31 endothelial integrity image at x 200 magnification.

Video S1. Assessment of lidocaine-releasing properties of the gel on the left sided femoral artery under surgically induced vasospasm.

Video S2. Arterial image obtained from CT angiography.

Video S3. Microsurgical process of femoral artery anastomosis in the gel medium.

Table S1. Molecular dynamics simulations system details

Model Name	Molecules	Box size (Å^3)	# ions	#water	Duration (ns)	#replica
Protonated lidocaine in $CaCl_2$ solution	10 protonated lidocaine	90 × 100 ×100	40 Ca^{2+}<br>90 Cl^{-1}	9250	50	2
Neutral lidocaine in $CaCl_2$ solution	10 neutral lidocaine	90 × 90 ×90	40 Ca^{2}<br>80 Cl^{-}	9250	50	2
PGAL and protonated lidocaine in water	12 protonated lidocaine<br>22-unit long poly-galacturonic acid chain with –COO^{-} groups	90 × 100 ×100	10 Na^{+}	9259	50	2

Table S2. Self-healing recovery (RS%), creep recovery (RC%) and tack results of the hydrogel.

Code	RS%	RC%	Energy of tackiness (N.s)
4P-2	100	45	0.94

Table S3. Comparison of anastomosis duration and early and first week patencies following anastomosis. All values were given in mean±SD.

**Parameters**	**Artery**		**Vein**	
Anastomosis Duration (minutes)	Gel (n=20)	Control (n=22)	Gel (n=20)	Control (n=22)
	20.6±4.26	21.77±4.79	22.32±6.03	29.07±10.27
Mann-Whitney U	*p=0.65*		*p=0.002*	
Early Perioperative Patency	94.5% (21/22)	%95 (19/20)	81.8% (18/22)	75% (15/20)
Chi-Square	*p=0.945*		*p=0.591*	
First Week Patency	Gel (n=7)	Control (n=6)	Gel (n=7)	Control (n=6)
	71.4% (5/7)	83.3% (5/6)	57.4% (4/7)	50% (3/6)
Chi-Square	*p=0.612*		*p=0.797*	

Table S4. Comparison of Artery and Vein Diameter Changes after and before anastomosis between Gel and Control Groups. All values were given in mean±SD.

**Vessel Diameter (mm)**	**Gel Group (n=22)**	**Control Group (n=20)**
Artery Before Anastomosis	0.62±0.12	0.61±0.16
Artery After Anastomosis	0.96±0.15	0.74±0.16
Wilcoxon	*p=0.001*	*p=0.005*
ANOVA	*p=0.001*	
Vein Before Anastomosis	0.74±0.20	0.78±0.32
Vein After Anastomosis	1, 14±0.40	0.87±0.23
Wilcoxon	*p=0.001*	*p=0.027*
ANOVA	*p=0.001*	

Table S5. Temporal Changes in Femoral Artery Diameter in Pre-gel and Gel Conditions

**Time Intervals**	**Mean difference**	**Std. Error**	**p**	**95% CI lower limit**	**95% CI upper limit**
0-5.minutes	-0.25	0.061	*0.005*	-0.44	-0.06
0-10. minutes	-0.36	0.061	*<0.001*	-0.55	-0.17
0-15. minutes	-0.49	0.061	*<0.001*	-0.68	-0.30
5-10. minutes	-0.11	0.061	0.53	-0.30	0.08
5-15. minutes	-0.24	0.061	*0.007*	-0.43	-0.05
10-15. minutes	-0.13	0.061	0.286	-0.32	0.06

ANOVA test between groups p-value < 0.001, followed by Bonferroni Post-hoc tests for within-group comparisons.

Table S6. Comparison of inflammatory response in arteries and veins at different times between gel and control groups. All values were given in mean±SD.

**Groups**		**Giant Cell Count(mean±SD)**		**CD68 (mean±SD)**		**Inflammatory Cell Count (mean±SD)**	
		**Artery**	**Vein**	**Artery**	**Vein**	**Artery**	**Vein**
1st Week	Gel	0.8±0.61	1.36±1.13	2±1.45	3±3.08	10.15±3.46	9.38±1.72
	Control	0.98±1.19	0.68±0.73	2.88+1.76	3.48±1.84	9.69±4.60	8.77±1.58
	ANOVA	*p=1*	*p=1*	*p=1*	*p=1*	*p=1*	*p=1*
6th Week	Gel	2.39±0.91	2.92±1.94	1.28±1.44	0.94 ±0.3	10.67±4.34	10.9±4.70
	Control	3.32±2.12	1.92±1.22	0.92±0.94	1.22±1.09	10.17±3.2	11.97±7.29
	ANOVA	*p=1*	*p=1*	*p=1*	*p=1*	*p=1*	*p=1*
6th Month	Gel	1.71±1.21	3.38±2.22	0.84±0.71	1.87±1.39	12.53±2.39	14.2±4.100
	Control	1.56±0.96	3.6±2.68	0.65±0.74	0.89±1.08	8.09±2.70	10.12±4.59
	ANOVA	*p=1*	*p=1*	*p=1*	*p=1*	*p=0.3410*	*p=1*

Table S7. Comparison of vascular fibrosis and wall integrity in arteries and veins at 6 months.

**Groups**		**Reticulin**		**EVG**	
		**Negative**	**Positive**	**Positive**	**Negative**
Artery	Gel	6	1	5	2
	Control	7	0	5	2
	Chi-Square	*p=0.29*		*p=1*	
Vein	Gel	8	0	5	3
	Control	4	2	4	2
	Chi-Square	*p=0.078*		*p=0.872*	

Table S8. Patency rates (%) in femoral arteries and veins.

Groups	Sixth Week				Sixth Month			
	**Number of samples**	**Artery Diameter (mm)**	**Artery Flow rate ($ml.s^{-1}$)**	**Vein Diameter (mm)**	**Number of samples**	**Artery Diameter (mm)**	**Artery Flow rate ($ml.s^{-1}$)**	**Vein Diameter (mm)**
Gel	7	1.18±0.3	0.34±0.2	1.16±0.32	8	1.44±0.18	1.37±0.22	0.56±0.2
Control	7	1.19±0.32	0.29±0.12	1.17±0.45	7	1.44±0.14	1.47±0.21	0.71±0.21
Normal	12	1.16±0.25	0.29±0.14	1.21±0.34	15	1.43±0.21	1.36±0.22	0.61±0.22
ANOVA		p=0.973	p=0.785	p=0.962		p=0.98	p=0.54	p=0.421

Table S9. Femoral artery and vein diameters measured by Doppler Ultrasound at the end of sixth week and sixth month. Femoral artery flow rates were also calculated in gel and control groups and were compared to their normal by examining the same animals' left femoral artery and vein. All values were given in Mean±SD

Groups	6th Week		6th Month	
	**Artery**	**Vein**	**Artery**	**Vein**
Gel	100	85.71	100	87.50
Control	100	100	100	100
Chi-Square	*p=1*	*p=0.299*	*p=1*	*p=0.333*

References

(1) Humphrey, W.; Dalke, A.; Schulten, K. VMD: Visual Molecular Dynamics. *J Mol Graph* **1996**, *14* (1), 33–38. https://doi.org/10.1016/0263-7855(96)00018-5

(2) Nurjaya, S.; Wong, T. Effects of Microwave on Drug Release Properties of Matrices of Pectin. *Carbohydr Polym* **2005**, *62* (3), 245–257. https://doi.org/10.1016/j.carbpol.2005.07.029

(3) Kocaaga, B.; Kurkcuoglu, O.; Tatlier, M.; Batirel, S.; Guner, F. S. Low- methoxyl Pectin–Zeolite Hydrogels Controlling Drug Release Promote *in vitro* Wound Healing. *J Appl Polym Sci* **2019**, *136* (24), 47640. https://doi.org/10.1002/app.47640

(4) Rodrigues, N. V. S.; Cardoso, E. M.; Andrade, M. V. O.; Donnici, C. L.; Sena, M. M. Analysis of Seized Cocaine Samples by Using Chemometric Methods and FTIR Spectroscopy. *J Braz Chem Soc* **2013**. https://doi.org/10.5935/0103-5053.20130066

(5) Kottke, D.; Lura, A.; Lunter, D. J.; Breitkreutz, J. Manufacturing and Characterisation of a Novel Composite Dosage Form for Buccal Drug Administration. *Int J Pharm* **2020**, *589*, 119839. https://doi.org/10.1016/j.ijpharm.2020.119839

(6) Wei, Y.; Nedley, M. P.; Bhaduri, S. B.; Bredzinski, X.; Boddu, S. H. S. Masking the Bitter Taste of Injectable Lidocaine HCl Formulation for Dental Procedures. *AAPS PharmSciTech* **2015**, *16* (2), 455–465. https://doi.org/10.1208/s12249-014-0239-z

(7) Zanetti Baú, R.; Dávila, J. L.; Komatsu, D.; Akira d'Avila, M.; Gomes, R. C.; Duek, E. A. de R. Influence of Hyaluronic Acid and Sodium Alginate on the Rheology and Simvastatin Delivery in Pluronic-Based Thermosensitive Injectable Hydrogels. *J Drug Deliv Sci Technol* **2023**, *88*, 104888. https://doi.org/10.1016/j.jddst.2023.104888

(8) Tsui, H.-W.; Wang, J.-H.; Hsu, Y.-H.; Chen, L.-J. Study of Heat of Micellization and Phase Separation for Pluronic Aqueous Solutions by Using a High Sensitivity Differential Scanning Calorimetry. *Colloid Polym Sci* **2010**, *288* (18), 1687–1696. https://doi.org/10.1007/s00396-010-2308-5

(9) Sanandiya, N. D.; Vasudevan, J.; Das, R.; Lim, C. T.; Fernandez, J. G. Stimuli-Responsive Injectable Cellulose Thixogel for Cell Encapsulation. *Int J Biol Macromol* **2019**, *130*, 1009–1017. https://doi.org/10.1016/j.ijbiomac.2019.02.135

(10) Bertsch, P.; Diba, M.; Mooney, D. J.; Leeuwenburgh, S. C. G. Self-Healing Injectable Hydrogels for Tissue Regeneration. *Chem Rev* **2023**, *123* (2), 834–873. https://doi.org/10.1021/acs.chemrev.2c00179

(11) Bercea, M.; Constantin, M.; Plugariu, I.-A.; Oana Daraba, M.; Luminita Ichim, D. Thermosensitive Gels of Pullulan and Poloxamer 407 as Potential Injectable Biomaterials. *J Mol Liq* **2022**, *362*, 119717. https://doi.org/10.1016/j.molliq.2022.119717

(12) Yang, R.; Huang, J.; Zhang, W.; Xue, W.; Jiang, Y.; Li, S.; Wu, X.; Xu, H.; Ren, J.; Chi, B. Mechanoadaptive Injectable Hydrogel Based on Poly(γ-Glutamic Acid) and Hyaluronic

Acid Regulates Fibroblast Migration for Wound Healing. *Carbohydr Polym* **2021**, *273*, 118607. https://doi.org/10.1016/j.carbpol.2021.118607

(13) Geng, Z.; Ji, Y.; Yu, S.; Liu, Q.; Zhou, Z.; Guo, C.; Lu, D.; Pei, D. Preparation and Characterization of a Dual Cross-Linking Injectable Hydrogel Based on Sodium Alginate and Chitosan Quaternary Ammonium Salt. *Carbohydr Res* **2021**, *507*, 108389. https://doi.org/10.1016/j.carres.2021.108389

(14) Hong, X.; Stegemann, J. P.; Deng, C. X. Microscale Characterization of the Viscoelastic Properties of Hydrogel Biomaterials Using Dual-Mode Ultrasound Elastography. *Biomaterials* **2016**, *88*, 12–24. https://doi.org/10.1016/j.biomaterials.2016.02.019

(15) Rial, R.; Liu, Z.; Ruso, J. M. Soft Actuated Hybrid Hydrogel with Bioinspired Complexity to Control Mechanical Flexure Behavior for Tissue Engineering. *Nanomaterials* **2020**, *10* (7), 1302. https://doi.org/10.3390/nano10071302

(16) Srivastava, N.; Roy Choudhury, A. Thermo-Reversible Self-Assembled Novel Gellan Gum Hydrogels Containing Amino Acid Biogelators with Antibacterial Activity. *Carbohydr Polym* **2024**, *324*, 121462. https://doi.org/10.1016/j.carbpol.2023.121462

(17) Ali, I.; Ali Shah, L.; Rehman, T. ur; Faizan, S. Investigation of the Viscoelastic Behavior of PVA-P(AAm/AMPS) IPN Hydrogel with Enhanced Mechanical Strength and Excellent Recoverability. *Journal of Polymer Research* **2022**, *29* (1), 7. https://doi.org/10.1007/s10965-021-02841-2

(18) Dolz, M.; Hernández, M. J.; Delegido, J. Creep and Recovery Experimental Investigation of Low Oil Content Food Emulsions. *Food Hydrocoll* **2008**, *22* (3), 421–427. https://doi.org/10.1016/j.foodhyd.2006.12.011

(19) Bhusari, S.; Hoffmann, M.; Herbeck-Engel, P.; Sankaran, S.; Wilhelm, M.; del Campo, A. Rheological Behavior of Pluronic/Pluronic Diacrylate Hydrogels Used for Bacteria Encapsulation in Engineered Living Materials. *Soft Matter* **2024**. https://doi.org/10.1039/D3SM01119D

(20) Sun, S.; Li, M.; Liu, A. A Review on Mechanical Properties of Pressure Sensitive Adhesives. *Int J Adhes Adhes* **2013**, *41*, 98–106. https://doi.org/10.1016/j.ijadhadh.2012.10.011

(21) Kim, S.-H.; Lee, S.-H.; Lee, J.-E.; Park, S. J.; Kim, K.; Kim, I. S.; Lee, Y.-S.; Hwang, N. S.; Kim, B.-G. Tissue Adhesive, Rapid Forming, and Sprayable ECM Hydrogel via Recombinant Tyrosinase Crosslinking. *Biomaterials* **2018**, *178*, 401–412. https://doi.org/10.1016/j.biomaterials.2018.04.057

(22) Bouras, R.; Mohand, C. S. I. H.; Sonebi, M. Adhesion and Rheology of Joints Fresh Mortars. *J Mater Eng Struct* **2019**, *6* (2), 157–165. https://doi.org/10.3933/ApplRheol-19-51970
